# Lipid oversupply induces CD36 sarcolemmal translocation via dual modulation of PKCζ and TBC1D1: an early event prior to insulin resistance

**DOI:** 10.7150/thno.40021

**Published:** 2020-01-01

**Authors:** Bili Zhu, Ming-Yue Li, Quanming Lin, Zhicheng Liang, Qihang Xin, Menghuan Wang, Zhendan He, Xiaomei Wang, Xuli Wu, George G. Chen, Peter CY Tong, Weizhen Zhang, Li-Zhong Liu

**Affiliations:** 1 Guangdong Provincial Key Laboratory of Regional Immunity and Diseases, Department of Physiology, Health Science Center, School of Medicine, Shenzhen University, Shenzhen, China; 2 Department of Surgery, Prince of Wales Hospital, The Chinese University of Hong Kong, Shatin, N.T., Hong Kong; 3 Key Laboratory of Natural Small Molecular Drugs, School of Pharmacy, Shenzhen University, Shenzhen, China; 4 School of Public Health, Shenzhen University, Shenzhen, China; 5 Jockey Club School of Public Health and Primary Care, Chinese University of Hong Kong, Prince of Wales Hospital, Shatin, Hong Kong; 6 Department of Physiology and Pathophysiology, Peking University Health Science Center, Beijing, China

**Keywords:** CD36, fatty acid, PKCζ, TBC1D1

## Abstract

Lipid oversupply may induce CD36 sarcolemmal translocation to facilitate fatty acid transport, which in turn causes dyslipidemia and type 2 diabetes. However, the underlying mechanisms of CD36 redistribution are still yet to be unraveled.

**Methods**: High fat diet fed mice and palmitate/oleic acid-treated L6 cells were used to investigate the initial events of subcellular CD36 recycling prior to insulin resistance. The regulation of CD36 sarcolemmal translocation by lipid oversupply was assessed by insulin tolerance test (ITT), oral glucose tolerance test (OGTT), glucose/fatty acid uptake assay, surface CD36 and GLUT4 detection, and ELISA assays. To elucidate the underlying mechanisms, specific gene knockout, gene overexpression and/or gene inhibition were employed, followed by Western blot, co-immunoprecipitation, immunostaining, and kinase activity assay.

**Results**: Upon lipid/fatty acid overload, PKCζ activity and TBC1D1 phosphorylation were enhanced along with increased sarcolemmal CD36. The inhibition of PKCζ or TBC1D1 was shown to block fatty acid-induced CD36 translocation and was synergistic in impairing CD36 redistribution. Mechanically, we revealed that AMPK was located upstream of PKCζ to control its activity whereas Rac1 facilitated PKCζ translocation to the dorsal surface of the cell to cause actin remodeling. Furthermore, AMPK phosphorylated TBC1D1 to release retained cytosolic CD36. The activated PKCζ and phosphorylated TBC1D1 resulted in a positive feedback regulation of CD36 sarcolemmal translocation.

**Conclusion**: Collectively, our study demonstrated exclusively that lipid oversupply induced CD36 sarcolemmal translocation via dual modulation of PKCζ and TBC1D1, which was as an early event prior to insulin resistance. The acquired data may provide potential therapy targets to prevent lipid oversupply-induced insulin resistance.

## Introduction

The prevalence of obesity as a global issue has provoked a concerning healthcare challenge. Obesity is closely associated with insulin resistance (IR), which predisposes the onset of type 2 diabetes (T2D) 1. The skeletal muscle plays a pivotal role in glucose homeostasis due to it accounting for up to 80%-90% of insulin-stimulated glucose disposal 2 and thus bearing considerable significance in the pathogenesis of insulin resistance 3. The inability of insulin to stimulate glucose uptake into skeletal muscle is one of the earliest hallmarks of type 2 diabetes (T2D) development, which is currently reaching epidemic proportions worldwide 4. One underlying rationale of skeletal muscle IR in obesity is fatty acid (FA) oversupply, which overwhelms the β-oxidation in mitochondria 5, 6. The overconsumption of lipids in skeletal muscle may cause ectopic accumulation of intramyocellular diacylglycerol 7, 8 and ceramide content 9. These accumulations may induce insulin resistance via protein kinases C family members 10, 11.

Skeletal muscle that are exposed to high lipid concentrations display an elevated rate of long-chain fatty acid (LCFA) uptake. In the past years, it has been established that LCFAs cross the plasma membrane via proteins within the myocytes. Chronic elevation of LCFA uptake requires a family of fatty acid transport proteins (FATP1-6, 63kDa), plasma membrane associated fatty acid-binding protein (FABPpm, 40kDa) and fatty acid translocase (CD36, 85-88KD). Out of these proteins, CD36 has been identified as a key LCFA transporter in the myocardium and skeletal muscle 12-15. CD36 is currently known to be a scavenger receptor protein (class B) superfamily member and is present in a variety of mammalian cell types. CD36 molecule is comprised of two transmembrane domains and a relatively large extracellular domain. Both the amino and carboxyl termini of CD36 are located within the cytoplasm 16-18. Through binding extracellular FABPpm 19 and intracellular FABPc 20, CD36 functions in sequestering fatty acids in the membrane. It helps to organize the fatty acids within specific membrane domains (presumably lipid rafts) for proper transportation and manufacturing to achieve subsequent transport and oxidization. CD36 possesses a key role in the alterations of lipid metabolism upon fatty acid oversupply, thus it has been implicated in the etiology of obesity-induced or high fat diet-induced ectopic lipid accumulation and insulin resistance. The significance of CD36 proposes it as a target for therapeutic intervention 15.

Not only is CD36 present at the sarcolemma, but also stored in intracellular membrane compartments. In response to insulin stimulation or muscle contraction, CD36 may translocate from endosomes to plasma membrane to increase LCFA uptake. The pattern is similar to that of glucose transporter 4 (GLUT4) translocation 15. In skeletal muscle, CD36 expression and/or subcellular distribution are/is altered during obesity and type 2 diabetes 21, 22. In high fat feeding rodent models, insulin resistance is exacerbated by accumulation of specific bioactive lipid species in skeletal muscle 23. The accumulation is caused by upregulation of sarcolemmal CD36 and fatty acid transport 24. In insulin-resistant rodent models, CD36 has been found responsible for the chronically elevated LCFA influx upon lipid overload. Studies in diabetic animals and humans with T2D indicate that rates of FA transport into muscle markedly increase due to permanent relocation of CD36 to the sarcolemma membrane. Furthermore, the increases in sarcolemma CD36 content correlated well with rates of FA transport in lean, obese and T2D muscle 13. All the experimental results indicate that abnormal distribution of CD36 on cell membrane is a precondition that mediates excess fatty acid uptake prior to insulin resistance. Thus, CD36 plays an essential role in the early stage development of insulin resistance due to lipid overload. However, there is limited information to illustrate the alteration of CD36 distribution pattern in skeletal muscle stimulated by high fat condition (excess fatty acid supply) while insulin sensitivity still persists.

The atypical protein kinase Cs (aPKCs) include PKCζ 25 and λ 26 (human PKCι and mouse PKCλ are orthologues). These two aPKCs can be interchangeable and are activated by insulin via phosphoinositide 3-kinase (PI-3K), and have been suggested to be required for insulin-stimulated GLUT4 translocation 27. PKCζ is further regulated by Rac1 to mediate the dynamic change of the actin structure, which in turn guides the docking/fusion of GLUT4 vesicles with plasma membrane 28, 29. It is unknown whether PKCζ could regulate CD36 translocation in a similar manner upon FA oversupply. On the other hand, the small GTPase Rac1 could be activated by exercise/muscle contraction and has been found necessary for insulin-stimulated GLUT4 translocation 30. The increment of Rac1 activity (Rac1-GTP binding level) is AMPK-independent during the process of exercise/muscle contraction 31. Furthermore, Rac1 exhibits its function in actin cytoskeleton remodeling with influence over membrane partitioning and thus membrane organization 32. Rac1 can be activated in pancreatic beta cells by PA addition, PA-induced superoxide generation and mitochondrial dysfunction 33. It is worthy of determining the legitimacy of Rac1-PKCζ-actin pathway involvement upon FA-induced CD36 translocation similar to that in insulin-induced GLUT4 translocation.

In muscle, two paralogue Rab-GTPase activating proteins (GAPs), namely TBC1D1 and TBC1D4 (also called AS160), are involved in both insulin-stimulated and AMPK-mediated translocation of GLUT4 from cytosol to plasma membrane, facilitating glucose transport 34. The phosphorylation signatures of TBC1D1 and TBC1D4 are different between insulin-stimulated and AMPK-mediated signal pathways 34, 35, suggesting their distinct roles in the regulation of muscle glucose transport as to insulin and exercise. AMPK, evidently activated by FA supply, is a conserved fuel-sensing enzyme universal to mammalian cells. The enzyme bears an important role in glucose and fatty acid metabolism for maintenance of ATP balance 36, 37. It is probable that either GAP or both are involved in AMPK-mediated CD36 translocation upon FA stimulation.

Current research aims to further decipher the signaling pathways, vesicular trafficking and cytoskeletal network that control the short-term subcellular recycling of CD36 in response to FA overload. Experimental outcomes reveal the propagation of signal transduction upon surface CD36 binding to extracellular FA. The cascade would lead to increased PKCζ activity and TBC1D1 phosphorylation, promoting CD36 sarcolemmal translocation and FA uptake.

## Results

### High-fat diet increased activity of AMPK/aPKCs and surface CD36 in skeletal muscle before the onset of insulin resistance

**Figure 1:**
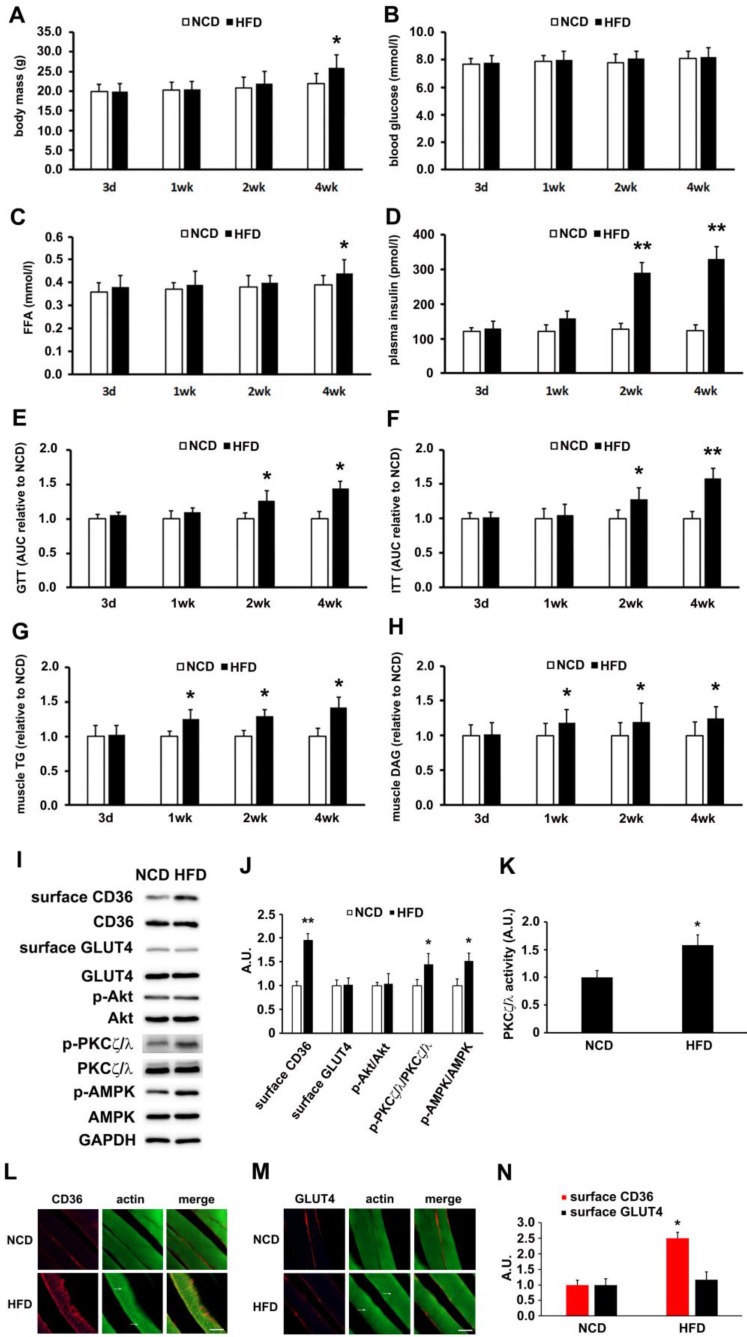
** HFD-induced changes of metabolic profiles and kinases activation. (A) Body mass. (B) Blood glucose. (C) Free fatty acid (FFA.; (D) Plasma insulin. (E) Glucose tolerance test (GTT). (F) Insulin tolerance test (ITT). (G) Skeletal muscle triglyceride (TG). (H) Skeletal muscle diacylglycerol (DAG). (I) Surface CD36/GLUT4 and kinases detection after three days of HFD-feeding.** Giant sarcolemmal vesicles were isolated from skeletal muscle of behind limbs and proteins were examined with antibodies as indicated**. (J) The quantitative analysis of western blot results.** The protein bands in Figure I were quantified. A value of 1 was assigned to the NCD control condition. The data was representative of 3 independent experiments. Data are means ± SE, * p<0.05 and ** p<0.01 vs NCD by t-test. **(K) PKCζ/λ activity.** The kinase activity was measured with specific immunoprecipitates from the 40 μg of giant sarcolemmal vesicles. The data is representative of 3 independent experiments. Data were means ± SE, * p<0.05 vs NCD by t-test. **(L) and (M) Immunostaining of CD36/GLUT4 and actin.** Skeletal muscle samples from NCD- and HFD- feeding mice (3d) were double stained for CD36/GLUT4(red) and actin(green) respectively. Bar, 10 μm. The images are representative of three experiments with tibialis anterior muscle and soleus muscle. **(N) Quantification of CD36/GLUT4 localization.** The stained CD36/GLUT4 (Red) was scanned and analyzed with Image J software. A value of 1 was assigned to the NCD group. Data are means ± SE, * p<0.05 vs NCD by t-test.

High-fat diet (HFD) fed mice were used to determine the lapse prior to glucose and insulin intolerance development. Significant divergence in mass occurred at the 4th week time point (Figure 1A). Blood glucose (Figure 1B) and free fatty acid (Figure 1C) exhibited significant elevation after 4 weeks of HFD feeding. Plasma insulin increased notably 2 weeks into HFD and persisted thereafter (Figure 1D). Glucose and insulin intolerance were evident after 2 weeks of HFD (Figure 1E and 1F). Skeletal muscle lipid content (TG and DAG) was not altered after 3 days of HFD feeding, but elevated after 1 week (Figure 1G-H). Obviously, impaired insulin sensitivity and glucose intolerance commenced at the second week of HFD feeding. It was seen that HFD-feeding mice provided us a model to explore the underlying mechanism of physiological change before and after the occurrence of insulin resistance.

Though HFD-feeding for 3 days (Figure 1) had no significant effect on glucose tolerance and insulin sensitivity (The insulin sensitivity was further confirmed by Tyr phosphorylation of IRS-1 and the association of PI3-kinase p85 subunit with IRS-1 (Figure S1)), it was unknown whether there had been alteration to the metabolism of fatty acid and glucose. Following that, we prepared muscle giant vesicles to detect possible distribution alters of CD36 and GLUT4 at this time point. Within the same hallmark, sarcolemmal CD36 of HFD group increased and the total CD36 protein level was not altered. Sarcolemmal GLUT4 distribution and GLUT4 expression remained unchanged (Figure 1I-J). We proceeded to explore the mechanism of CD36 translocation at this time point. Both PKCζ/λ and Akt were distal targets in insulin signal pathway that mediated insulin-induced CD36 and GLUT4 translocation, coordinating fatty-acid and glucose uptake respectively. It was worthy of investigating the roles of these two kinases upon HFD feeding. It was found that Akt phosphorylation (Akt activity) and expression were not altered (Figure 1I-J). However, PKCζ/λ phosphorylation (Figure 1I-J) and activity (Figure 1K) were increased by HFD. Thus, PKCζ/λ phosphorylation could be used to mark its activity, which was similar to the condition of insulin treatment 38. Given the evidence that AMPK could be involved in fatty acid metabolism 39, we also detected AMPK activity simultaneously. Upon HFD feeding, the activity of AMPK increased significantly (Figure 1I-J). In consistence with the western result of surface CD36 and GLUT4 detection, immunostaining of CD36 and GLUT4 in skeletal muscle tissue of NCD mice indicated that small amounts of CD36 and GLUT4 existed on the sarcolemma at the 3-day time point (Figure 1L-M). HFD-feeding for 3 days led to an increase of CD36 but not GLUT4 on sarcolemma (Figure 1L-N). HFD induced actin structure distortions, which reflected disorientations in the skeletal muscle cross-striated arrangement (Figure 1I-J, as white arrow head indicated). Results of Figure 1 demonstrated that HFD-stimulated sarcolemmal CD36 redistribution was a predisposing event to the impairment of insulin sensitivity. AMPK, PKCζ/λ and actin were involved in the mentioned process.

### Palmitate treatment increased activity of AMPK/aPKCs and surface CD36 in L6 myotubes before occurrence of insulin resistance

**Figure 2:**
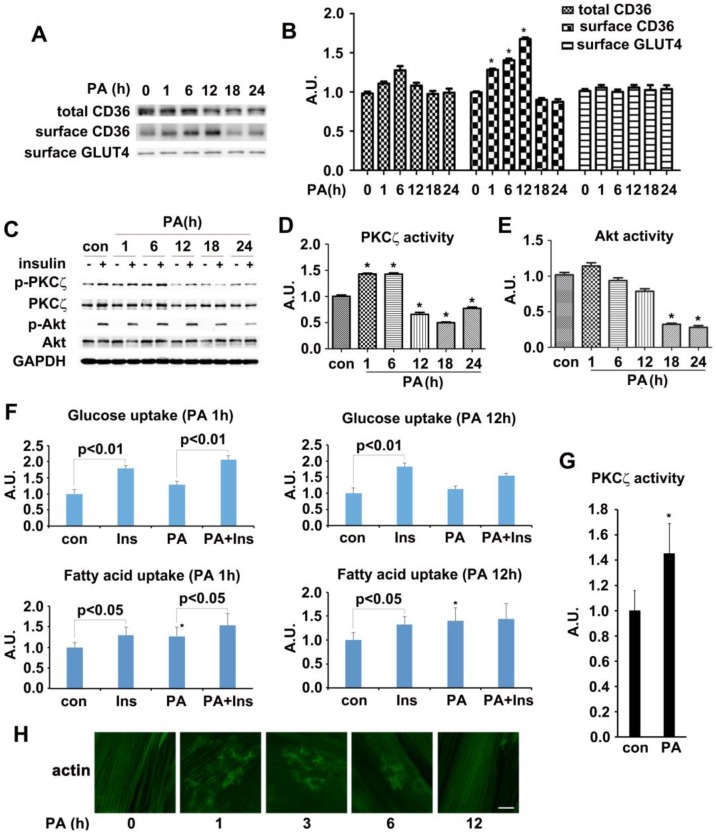
** Effect of palmitate on kinases activity, CD36/GLUT4 distribution, FA/glucose uptake and actin structure. (A) Time-course assay of CD36 expression after palmitate treatment.** Cells were left untreated or treated with 500 μmol/L PA (mol ratio of palmitate and albumin was 3:1) for different timeframes to prepare the total protein and surface proteins*.* Samples were immunoblotted with indicated antibodies**. (C) Effect of palmitate on insulin-induced kinases activity.** Cells were left untreated or treated with PA for different timeframes followed by adding insulin and incubation for another 10 min. The total protein was extracted and immunoblotted with indicated antibodies. **(B), (D) and (E) The quantitative analysis of western blot results.** The protein bands in A and C were quantified. A value of 1 was assigned to the control condition. Data are means ± SE (n=3), * p<0.05 vs. 0 h or * p<0.05 vs. con by t-test. **(F) Glucose assay and FA uptake assay.** After myotubes were treated with PA for 1 h or 18 h, insulin was added for another 10 min and used for glucose and FA uptake. Data were means ± SE (n=3), * p<0.05 vs. con by t-test. **(G) Measurement of PKCζ activity.** The activity was measured with its immunoprecipitates from the 500 μg of whole cell lysates. Data were means ± SE (n=3), * p<0.05 by t-test. **(H) Immunostaining of actin in cells after PA treatment.** Cells treated with PA for different times were fixed and stained for actin (green), Bar, 10 μm. The images are representative of three experiments.

The *in vivo* experimental data indicated that CD36 had translocated to sarcolemma before the onset of insulin resistance in HFD fed mice. We then attempted to reillustrate the same response to fatty acid oversupply *in vitro*. Differentiated L6 skeletal muscle cells (myotubes) were treated with palmitate (PA) for different times. Time course experiment showed that CD36 expression level increased significantly within 6 h but dropped after 18h (with no significant difference) in presence of PA (Figure 2A-B). It was notable that the morphology of myotubes worsened and quite the number of myotubes detached from the bottom of culture vessels after PA treatment for 18h, indicating the effect of lipid toxicity and eventual apoptosis of cells. In this circumstance, the translocation of CD36 to plasma membrane was impaired (Figure 2A). It was obvious that CD36 kept translocating to plasma membrane after 1 h of PA stimulation and attained peak at 12 h, declining occurred thereafter. Despite of PA treatment, the content of surface GLUT4 remained unchanged (Figure 2A-B). We next explored whether PA treatment impacted insulin sensitivity. Myotubes were first incubated with PA for different times and then stimulated by insulin. PKCζ, which was predominate aPKC in L6 skeletal muscle cells, related positively to Thr410 phosphorylation in response to insulin stimulation 38, 40. Moreover, PKCζ and λ were interchangeable in carrying out their biological functions 27. Based on the above facts, we mainly focused on PKCζ for the remaining experiments. Both PKCζ and Akt phosphorylation were detected as markers indicating insulin sensitivity. 12h PA treatment blocked insulin-stimulated PKCζ activation (Figure 2C-D). When myotubes were incubated with PA for 18 h, insulin-stimulated Akt activation became weaker and its activity diminished after 24 h (Figure 2C and 2E). A notable result was obtained with 1h PA treatment, where PKCζ activated significantly. However, it should be noted that PA itself had no effect on Akt activation. The above results showed that short lasting (within 6h) incubation with PA would not affect insulin signal transduction. Insulin sensitivity was impaired after prolonged PA treatment (>12h). To further demonstrate the interference of PA on insulin signal transduction, insulin-stimulated FA and glucose uptake were measured (Figure 2F). Pre-treatment with PA for 1h would not influence insulin-stimulated glucose and FA uptake. Although PA alone enhanced FA uptake notably, it had no effect on glucose uptake, yet its joint efforts with insulin could produce an additive effect on FA uptake. When myotubes were treated with PA for 12 h, insulin-stimulated FA and glucose uptake were inhibited. Intriguingly, PA still caused a remarkable FA uptake even though insulin enhanced FA uptake had been impaired. Obviously, the mechanism of PA induced FA uptake was different in comparison with insulin. It was shown that short-term PA treatment enhanced PKCζ activity (Figure 2G) and its phosphorylation (Figure 2C-D), these results were consistent with *in vivo* outcomes, indicating that phosphorylation of PKCζ could be used as a marker to label its activity in presence of PA *in vitro*. Previously, we found that PKCζ could mediate insulin effect on glucose uptake via actin remodeling 28, thus further evaluation on PA influence over actin morphology was conducted. Short-term incubation with PA (≤6h) induced rearrangement of actin structure in a way similar to insulin stimulated actin remodeling. However, the dynamic change of actin remodeling disappeared 12h later, and actin morphology returned to its initial form as stress fibers (Figure 2H). Based on the above findings, 1h PA incubation was employed for all further experiments to explore the molecular mechanism of PA-induced CD36 translocation and FA uptake prior to insulin resistance in L6 myotubes. Presence of insulin sensitivity post one hour PA treatment was confirmed by IRS-1 Tyr phosphorylation and association of PI3-kinase p85 subunit with IRS-1 (Figure S1). In order to clarify that PKCζ activation, CD36 relocation and FA uptake (Figure 2F) were purely caused by PA, L6 myotubes were first treated with PA, insulin and PA+insulin respectively to measure surface CD36 and PKCζ activity. In contrast to the function of PA and insulin alone, PA+insulin exhibited an additive effect on PKCζ activation and CD36 sarcolemmal translocation (Figure S2). Application of chemical inhibitors (SSO for PA, compound C (C.C) for AMPK, Wortmannin (WM) for PI3-kinase and pseudosubstrate (PS) for PKCζ) revealed that: 1) PA and insulin induced FA uptake via different pathways (PA worked through AMPK and insulin through PI3K) with PKCζ being the crosstalk; 2) insulin action could be ruled out during the process of PA stimulation (1h) (Figure S2).

### Palmitate induced signal transduction via its binding to surface CD36

**Figure 3:**
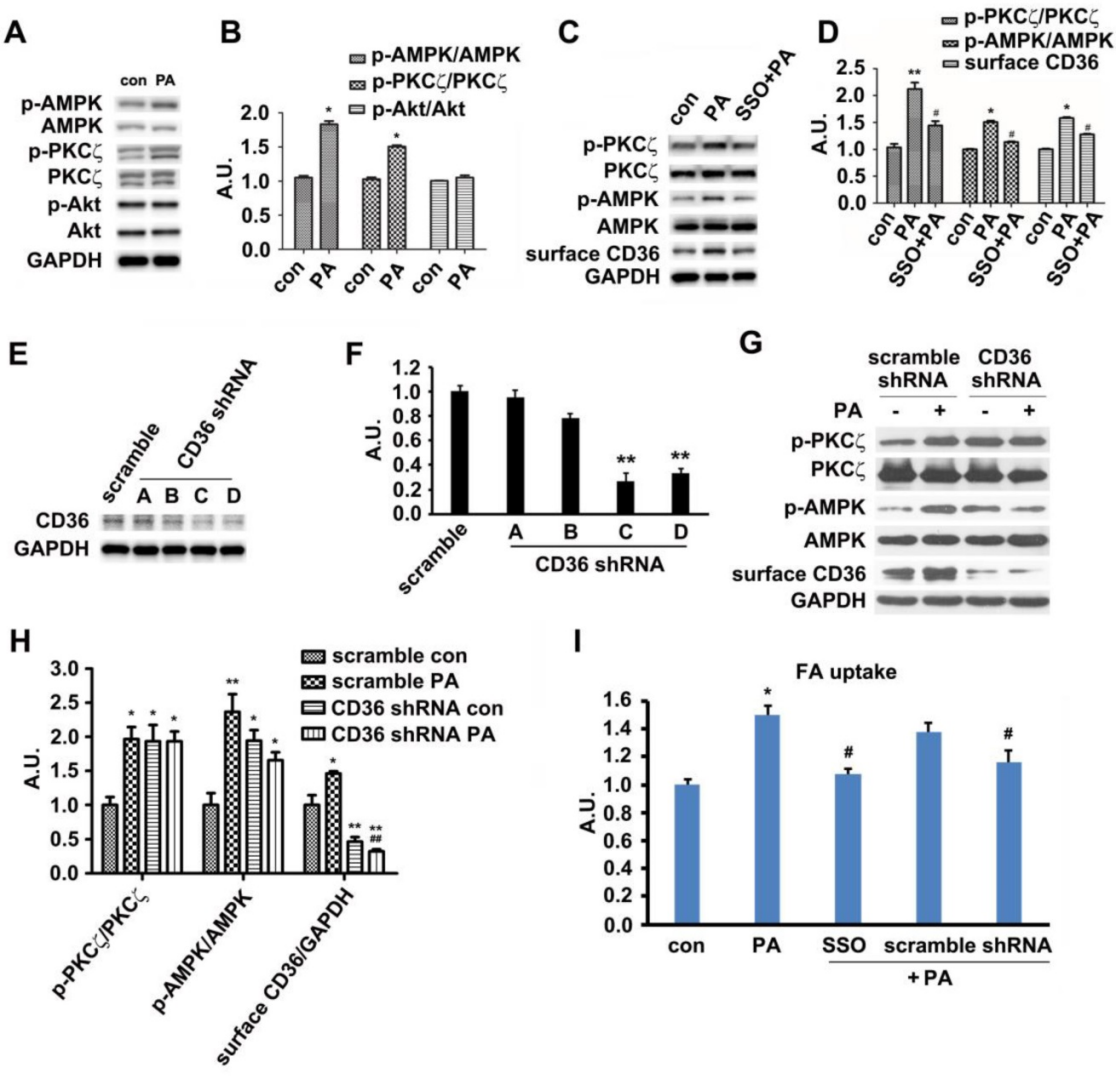
** Palmitate induced signal transduction via surface CD36. (A) Effect of PA on kinase activation; (C) Inhibition effect of SSO.** Cells were left untreated or treated with 500 μmol/L PA for 1 h; or cells were pretreated with 200 μM SSO for 20 min, followed by addition of 500 μmol/L PA and incubation for another 1 h. Then whole cell lysis proteins were immunoblotted with indicated antibodies. **(E) Assessment of CD36 shRNA.** Cells were transfected with CD36 shRNAs respectively and maintained for another 48 h. Whole cell lysis protein was immunoblotted with indicated antibodies. **(G) Effect of CD36 knockdown on kinase activation and surface CD36.** Cells were first transfected with scramble and CD36 shRNAs respectively followed by PA treatment. Whole cell lysis protein was immunoblotted with indicated antibodies.** (B), (D), (F) and (H) The quantitative analysis of western blot results.** The protein bands in A, C, E and G were quantified. A value of 1 was assigned to the control. Data were means ± SE (n=3), *p<0.05, **p<0.01 vs. con, or ^#^*p*<0.05, ^##^*p*<0.01 vs. PA, by t-test. **(I) FA uptake assay.** Cells were pre-treated with chemicals respectively followed by PA treatment as before, then FA uptake was measured. Data were means ± SE (n=5), *p<0.05 vs. con or ^#^*p*<0.05 vs. PA by t-test.

*In vivo* results exhibited that HFD-feeding for 3 days would not impair glucose tolerance and insulin sensitivity. At this time point, HFD-feeding increased AMPK and PKCζ activity along with CD36 surface translocation. Akt activity and sarcolemmal GLUT4 were not affected by the diet. Thus, we followed up by examining the effects of PA on AMPK/PKCζ/Akt activity in myotubes. The experimental results showed that PA (1 h) could enhance AMPK and PKCζ activity while not affecting Akt (Figure 3A-B). Results from *in vitro* L6 myotubes were consistent with that of *in vivo* as shown in Figure 1. Both *in vitro* and *in vivo* experimental results demonstrated that short lasting FA oversupply could induce a signal transduction that led to AMPK/PKCζ activation inside cells. During the mentioned process, insulin sensitivity and insulin-induced glucose uptake persisted. Since cell surface CD36 could bind with FA and help its transportation, it was plausible that CD36 might mediate the signal transduction to regulate relevant FA metabolism. SSO, a membrane-impermeable sulfo-N-hydroxysuccinimidyl (NHS) ester of oleate, was a FA analogue. SSO irreversibly bound to CD36 and has been widely used to inhibit CD36-dependent FA uptake 41. As shown in Figure 3C and 3D, PA-induced activation of AMPK and PKCζ was blocked by SSO, accompanied with the elimination of PA-induced CD36 distribution on plasma membrane. Consistently, PA-stimulated FA uptake was inhibited as well (Figure 3I). To further demonstrate the involvement of CD36 in PA-induced signal transduction, CD36 expression was knocked down by its shRNAs. Out of the four tested CD36 shRNAs , number C and D came up with more prominent inhibitory effect (Figure 3E-F). When CD36 shRNA (number C) was transfected into the cells, PA-induced activation of PKCζ/AMPK was blocked (Figure 3G-H). Blockage of FA uptake by CD36 shRNA was similar to SSO (Figure 3I). These results showed that binding of PA with cell surface CD36 led to the activation of AMPK and PKCζ, promoting CD36 sarcolemmal translocation as well as FA uptake. It was worthy of noticing that CD36 knockdown could increase PKCζ/AMPK activity in absence of PA (Figure 3G-H), indicating the negative regulating role of CD36 on PKCζ/AMPK activation.

### Palmitate-induced signal transduction via CD36-Fyn-AMPK-PKCζ axis

**Figure 4:**
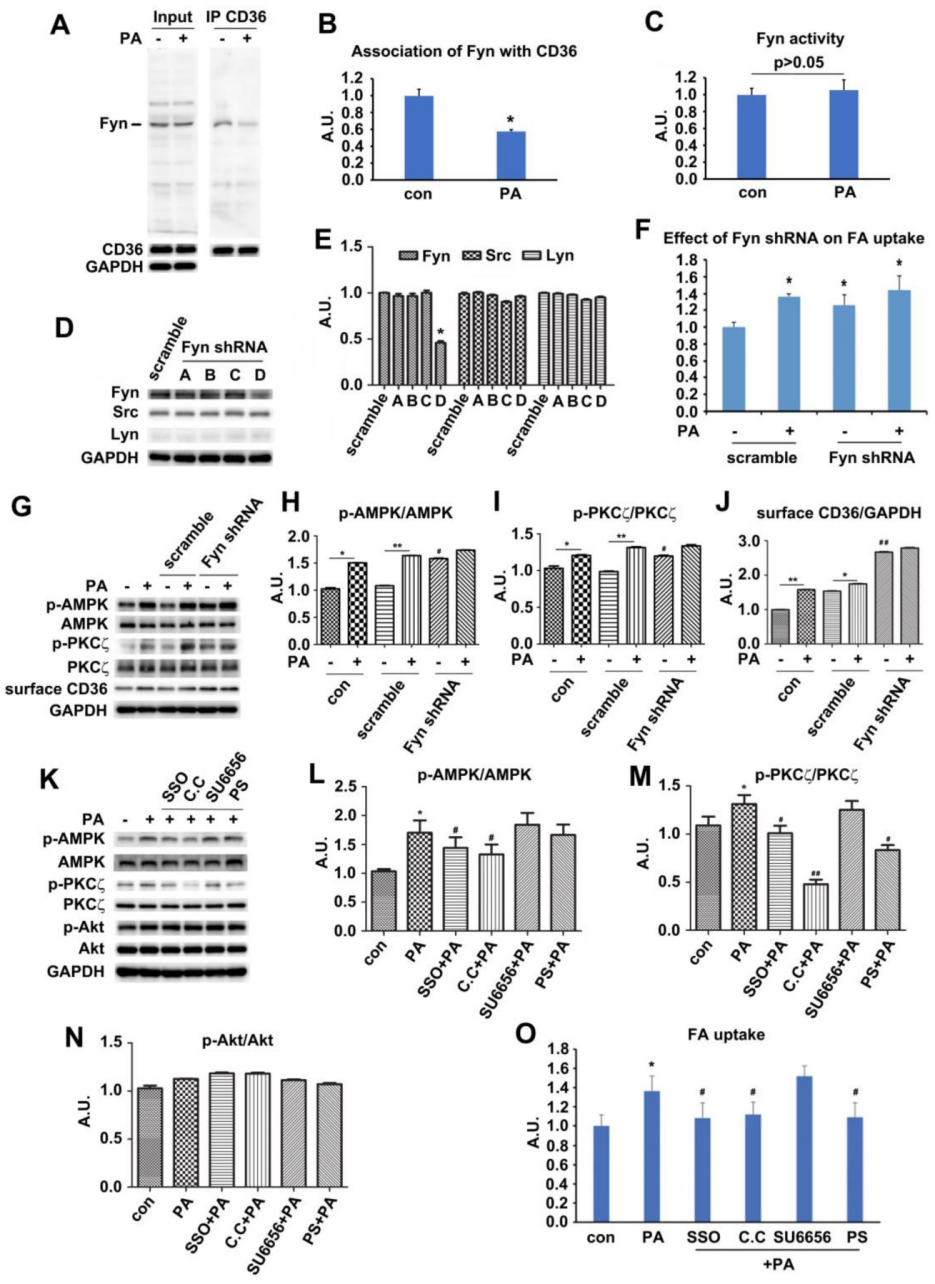
** Palmitate-induced signal transduction via CD36-Fyn-AMPK-PKCζ axis. (A) Association of Fyn with CD36.** Total cell lysis (input) was detected with indicated antibodies. CD36 immunoprecipitates (IP by CD36 antibody) were examined with CD36 and Fyn antibodies respectively. **(C) Fyn activity assay.** Cells were left treated or treated with 500 μmol/L PA for 1 h. Fyn was immunoprecipitated from the total cell lysis and analyzed with Tyrosine Kinase Activity Assay Kit. Data were means ± SE (n=4) and calculated by t-test. **(D) Assessment of Fyn shRNA.** Cells were transfected with Fyn shRNAs and maintained for another 48 h. Whole cell lysis protein was immunoblotted with indicated antibodies. **(G) Effect of Fyn on PA-induced kinases activity.** Cells were treated as indicated and whole cell lysis protein was immunoblotted with indicated antibodies. **(B), (E), (H), (I), (J), (L), (M) and (N) The quantitative analysis of western blot results.** The protein bands were quantified. A value of 1 was assigned to the control. Data were means ± SE (n=3), *p<0.05 vs. con for (B); *p<0.05 vs. scramble for (E); *p<0.05 or **p<0.01 vs. con/scramble (non-PA treatment), ^#^*p*<0.05 or ^##^*p*<0.01 vs. scramble (non-PA treatment) for (H), (I) and (J); *p<0.05 vs. con, ^#^*p*<0.05 or ^##^*p*<0.01 vs. PA for (M) and (N); by t-test. **(F) and (O) FA uptake assay.** Cells were transfected with shRNAs or pre-treated with chemicals respectively followed by PA treatment as before, then FA uptake was measured. Data were means ± SE (n=5), *p<0.05 vs. con or ^#^*p*<0.05 vs. PA, by t-test.

Since PA could induce signal transduction via surface CD36, we next explored how CD36 triggered this signal upon PA supply. Fyn has been reported to be associated with surface CD36 42. It also played important roles in mediating insulin signaling pathway 43, 44, and affected insulin sensitivity negatively 45. The Fyn protein level persisted in both basal and PA-stimulated condition while the association of Fyn with CD36 dropped in the presence of PA (Figure 4A-B). The results were consistent with Samovski's report 46. We further revealed that the disassociation of Fyn from CD36 did not bring negative effects on Fyn activity (Figure 4C). To further explore the functions of Fyn, four Fyn shRNA were tested to knock down Fyn expression, with shRNA number D showing efficient inhibition (Figure 4D-E). This knockdown of Fyn by shRNA was specific since it had no effect on the expression of other two Src family members-Src and Lyn (Figure 4D-E). Even when Fyn was knocked down by its shRNA (number D) in absence of PA, significant activation of both AMPK and PKCζ and elevation of surface CD36 were detected. The activity of AMPK and PKCζ along with surface CD36 increased slightly upon PA addition (Figure 4G-J). FA uptake assay indicated that inhibition of Fyn expression by its specific shRNA brought about more FA uptake, mimicking the effect of PA treatment. PA could not stimulate further FA uptake when Fyn was knocked down (Figure 4F). Mechanically, Fyn knockdown or chemical inhibition by SU6656 47 could sequester more LKB1 in cytosol, which in turn activated AMPK (Figure S3 A-D). Consistently, FA uptake was promoted when Fyn was inhibited (Figure 4F). Thus, the effect of CD36 knockdown was similar to the binding of PA with surface CD36 and Fyn knockout, disassembling CD36-Fyn complex and finally leading to AMPK and PKCζ activation. Since Fyn activity was not altered by PA treatment (Figure 4C), it was probable that Fyn disassociation from CD36 changed its cellular distribution and/or molecular structure, which in turn liberated restrictions on LKB1 distribution in nucleus.

The above data showed that PA stimulation could activate both AMPK and PKCζ through Fyn. We next investigated the hierarchy relation between AMPK and PKCζ. When the binding sites of FA on CD36 molecules were inhibited by SSO, PA-induced activation of AMPK and PKCζ was blocked. Src inhibitor SU6656 did not alter the effect of PA remarkably. Not only did AMPK specific inhibitor compound C (C.C) block PA-induced activation of AMPK but also inhibit PKCζ activity. However, pretreatment with PKCζ specific inhibitor pseudosubstrate (PS) inhibited PKCζ activity without elimination of PA-induced AMPK activation (Figure 4K-N). Intriguingly, all chemical reagents including PA had no effect on Akt activity (Figure 4K and 4N). The results indicated that AMPK was upstream of PKCζ and controlled its activation upon PA stimulation. Akt was uninvolved in PA induced signal transduction. Consistently, SSO, C.C and PS inhibited PA-induced fatty acid uptake, whereas SU6656 had no evident effect. Furthermore, there was no additive effect of FA uptake upon PA stimulation with SU6656 pretreatment (Figure 4O). It has been reported that SU6656, the Src kinase inhibitor, could affect AMPK activity 48. Experimental results with SU6656 application were similar to that of Fyn knockdown (Figure 4D-J, and Figure S1), suggesting that SU6656 would enhance phosphorylation of downstream targets. It was probable due to Thr172 phosphorylation lifetime being sufficient for dissociation of the inhibitor and subsequent catalysis prior to its dephosphorization 48. The data indicated that the FA binding to surface CD36 was the precondition for FA-induced signal transduction and FA uptake, involving AMPK and PKCζ in the process. Joint results with those in Figure 1 and Figure 3 proved that AMPK and PKCζ could be activated by PA stimulation, with AMPK locateing upstream of PKCζ.

### PKCζ mediated CD36 translocation and fatty acid uptake via actin remodeling

**Figure 5:**
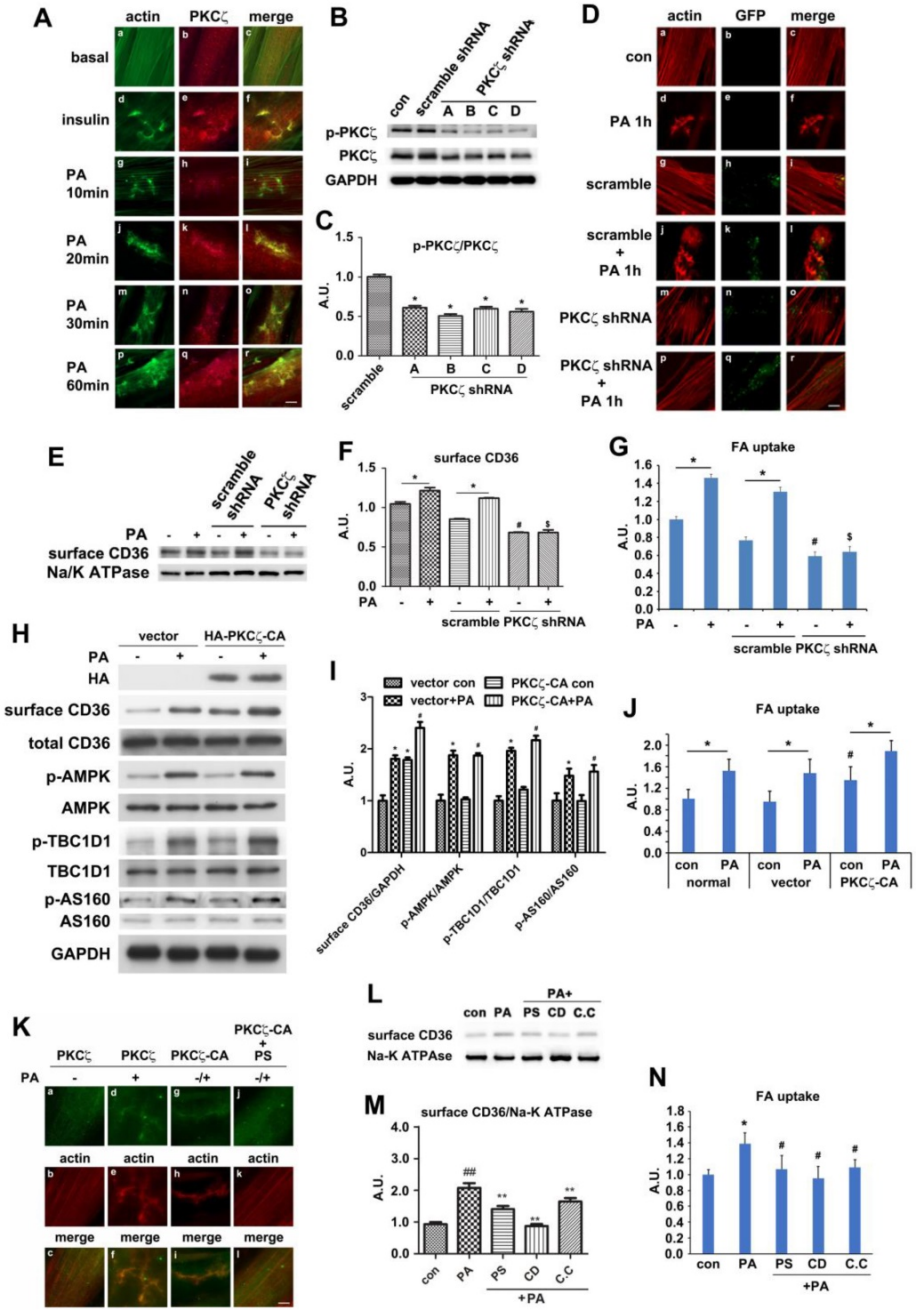
** PKCζ mediated PA-induced CD36 translocation and FA uptake via actin remodeling. (A) PKCζ distribution and its co-localization with actin.** Cells were treated with 100nmol/L insulin for 10 min or 500 μmol/L PA for different times as indicated. They were then stained to examine actin (green) and PKCζ (red). Bar, 10 μm. The images are representative of three experiments. **(B) Assessment of PKCζ shRNA and (C) Quantitative analysis of western blot results.** Cells were transfected with PKCζ shRNAs and maintained for 48h. Whole cell lysis protein was immunoblotted with indicated antibodies. Data were means ± SE (n=3), *p<0.05 vs. scramble by t-test. **(D) Inhibition of PA-induced actin remodeling by PKCζ shRNA.** Cells were left untreated or treated with 500 μmol/L PA for 1 h; or cells were transfected with scramble/PKCζ shRNAs respectively and maintained for 48 h, followed by the treatment of 500 μmol/L PA or not for 1 h. Then actin was stained to examine the actin structure change (red). The green dot indicated successful transfection and co-expression of GFP gene. Bar, 10 μm. The images are representative of three experiments. **(E) Effect of PKCζ knockdown on CD36 distribution and (F) The quantitative analysis of western blot results.** shRNAs were transfected into the cells followed by PA treatment or not. Whole cell lysis protein was immunoblotted with indicated antibodies. Data were means ± SE (n=3), *p<0.05 vs. con, ^#^*p*<0.05 vs. scramble con, ^$^*p*<0.01 vs. scramble PA, by t-test. **(G) Effect of PKCζ knockdown on FA uptake.** shRNAs were transfected into the cells followed by PA treatment or not. Then FA uptake was measured. Data were means ± SE (n=3), *p<0.05, ^#^*p*<0.05 vs. scramble con, ^$^*p*<0.01 vs. scramble PA, by t-test. **(H) Effect of PKCζ-CA overexpression and (I) Quantitative analysis of western blot results.** Cells were transfected with empty vector or vector contained PKCζ-CA. Proteins were analyzed with indicated antibodies after 48h of transfection. Data were means ± SE (n=3), *p<0.05 vs. vector con, ^#^*p*<0.05 vs. PKCζ-CA con, by t-test. **(J) Effect of PKCζ-CA on FA uptake.** Cells were transfected with empty or PKCζ-CA containing vectors and followed by PA treatment or not. Then FA uptake was measured. Data were means ± SE (n=3), *p<0.05, ^#^*p*<0.05 vs. vector con, by t-test. **(K) PKCζ controlled actin remodeling.** Vectors contained HA-tagged PKCζ-CA gene were introduced to the cells, and cells were maintained for another 48 h. Then, cells were treated with 10uM PS or not and stimulated with or without 500 μmol/L PA for 1 h at 37°C. Afterwards, the cells were double stained for endogenous PKCζ or HA-tag (green) and actin (red). Bar, 10 μm. The images were representative of three experiments. **(L) Surface CD36 detection and (M) Quantitative analysis of western blot results.** Cells were pre-treated with 10 μmol/L PKCζ inhibitor (PS), 1 μmol/L cytochalasin D (CD), 20 μM Compound C (C.C), respectively for 20 min, then treated with 500 μmol/L PA and incubated for another 1 h. Proteins were examined as indicated. ^##^*p*<0.01 vs. con, **p<0.01 vs. PA, by test. The data was representative of 3 independent experiments. **(N) Effect of chemicals on FA uptake.** Cells were treated with chemicals as before and FA uptake was measured. ^#^*p*<0.05 vs. con, *p<0.05 vs. PA, by test. The data was representative of 3 independent experiments.

The downstream position of PKCζ to AMPK along with its indispensable role in PA-induced CD36 translocation and fatty acid uptake inspired further investigation on PKCζ biological function. We have ever reported that PKCζ mediated insulin effect on GLUT4 translocation and glucose uptake via actin remodeling 28. We also showed that PA could induce actin rearrangement (Figure 2H) with morphology similar to insulin-induced actin remodeling. It was possible that PKCζ might also mediate PA effect via the dynamic change of actin structure. The immunostaining experiment exhibited that PA stimulation for distinct timeframes (within 1h) might cause actin remodeling (Figure 5A, g-r) similar to that of insulin stimulated (Figure 5A, d-f). At the same time, PKCζ translocated to the dorsal surface of cells, showing actin-remodeling like redistribution and co-localization with newly formed actin (Figure 5A). To further demonstrate the involvement of PKCζ in regulating actin structure, PKCζ specific shRNA were transfected into cells. Western blot experiment showed that PKCζ expression was inhibited efficiently (Figure 5B-C). When PKCζ shRNA (number D) was transfected into cells, PA-induced actin remodeling diminished (Figure 5D, m-r) in comparison with control (Figure 5D, a-f) and scramble shRNA (Figure 5D, g-l). PA-induced sarcolemmal CD36 translocation (Figure 5E-F) and FA uptake (Figure 5G) were inhibited as well.

When HA-tagged constitutively active PKCζ (PKCζ-CA) was transfected into cells, surface CD36 and FA uptake were enhanced in absence of PA. PA treatment further increased surface CD36 and FA uptake (Figure 5H-J). However, PKCζ-CA transfection left AMPK activity as well as TBC1D1 and AS160 phosphorylation state unaffected (Figure 5H-I). In contrast to PA-triggered actin remodeling and endogenous PKCζ distribution (Figure 5K, a-f), PKCζ-CA transfection could cause actin remodeling despite of PA treatment (Figure 5K, g-i). It should be noted that PS blocked effects of PKCζ-CA (Figure 5K, j-l).

To further demonstrate PKCζ and actin involvement in PA-induced CD36 translocation, cells were pre-treated with cytochalasin D (CD, which capped the plus end of the actin and inhibited actin polymerization), PKCζ inhibitor (PS), and AMPK inhibitor (C.C) respectively prior to PA stimulation. PA-induced surface CD36 increase was eliminated (Figure 5L-M). In consistence with the CD, C.C and PS effect on CD36 translocation, these chemicals could eliminate PA-induced fatty acid uptake (Figure 5N). The relationship between CD36 and actin was also examined. Similar to PKCζ distribution (Figure 5A, d-f) and insulin stimulation (Figure S4A, d-f), PA treatment for different time periods (within 1h) might cause CD36 translocation to plasma membrane and subsequent co-localization with actin remodeling (Figure S4A, g-r). To demonstrate that PKCζ-regulated dynamic change of actin structure was involved in CD36 re-distribution, immunostaining was employed. The experiment aimed to show CD36 distribution and its relationship with PKCζ and actin. PKCζ activity was blocked by its inhibitor PS during the process. When cells were pre-treated with PS, PA-induced CD36/PKCζ translocation to plasma membrane and their co-localization were inhibited (Figure S4B). Similarly, the co-localization of CD36 or PKCζ with actin at the dorsal surface of the cells were blocked (Figure S4C-D). These results indicated that PKCζ caused actin remodeling upon PA treatment, and this dynamic actin change might guide the insertion of cytosolic CD36 into plasma membrane where the actin remodeling occurred.

### Rac1 was activated by palmitate and regulated PKCζ distribution

**Figure 6:**
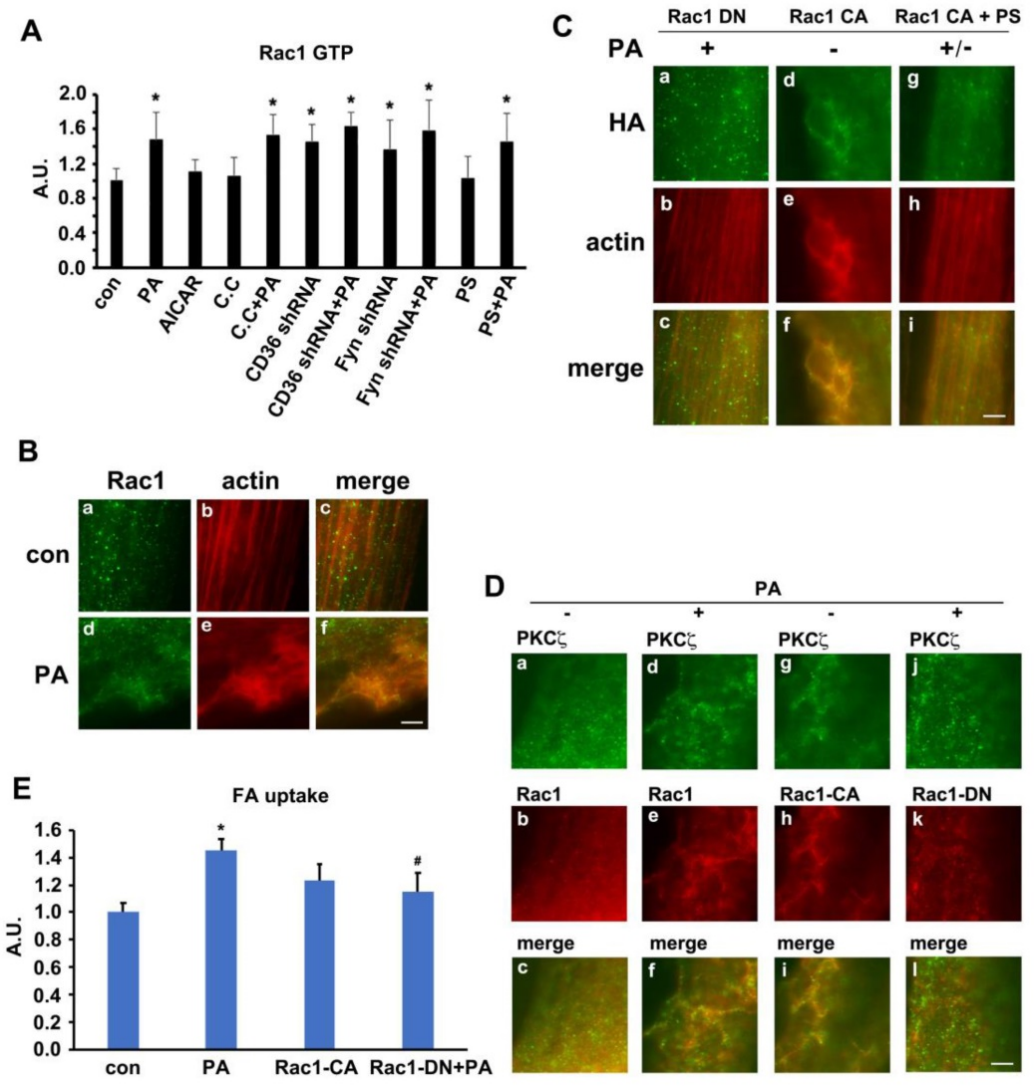
** Rac1 was activated by PA and regulates PKCζ distribution. (A) Rac1 could be activated by PA.** Cells were left untreated or treated with 500 μmol/L PA or 2 mmol/L AMPK activator AICAR respectively for 1 h; or cells were pretreated with 20 μM C.C, 10 μmol/L PS for 20 min followed by adding 500 μmol/L PA and incubation for another 1 h; or cells were transfected with CD36 or Fyn shRNA for 48 h, then cells were left untreated or treated with 500 μmol/L PA for 1 h. The Rac1 activity was measured according to instruction of the kit. Data were means ± SE (n=3), *p<0.05 vs. con by t-test. **(B) PA-induced co-localization between Rac1 and actin.** Cells were left untreated or treated with 500 μmol/L PA for 1h, then stained to examine Rac1 (green) and actin (red). Bar, 10 μm. The images are representative of three experiments. **(C) Rac1 controled actin remodeling.** HA-tagged Rac1-CA and Rac1-DN gene were introduced to the cells, cells were stimulated with or without 500 μmol/L PA for 1 h at 37°C after 48 h, or cells were pre-treated with 10 μmol/L PS for 20 min followed by adding 500 μmol/L PA and incubation for another 1 h. Then cells were double stained for HA-tagged Rac1-CA or Rac1-DN (green) and actin (red). Bar, 10 μm. The images were representative of three experiments. **(D) Rac1 could control PKCζ distribution.** Plasmids contained HA-tagged Rac1-CA and Rac1-DN gene were introduced to the cells with X-treme reagent, and cells were maintained for another 48 h. Then, cells were stimulated with or without 500 μmol/L PA for 1 h at 37°C. Afterwards, the cells were double stained for endogenous PKCζ (green) and Rac1 (red), or HA-tag for transfected Rac1 CA (red) and Rac1 DN (red) by HA-tag antibody. Bar, 10 μm. The images were representative of three experiments. **(E) Rac1 was required for PA-induced FA uptake.** HA-tagged Rac1-CA and Rac1-DN gene were introduced to the cells and after 48 h, cells were stimulated with or without 500 μmol/L PA for 1 h at 37°C followed by the measurement of FA uptake. Data were means ± SE (n=3), *p<0.05 vs. con, ^#^*p*<0.05 vs. PA, by t-test.

We have ever reported that Rac1, one of the GTPase molecules, played an important role in mediating insulin-induced actin remodeling via PKCζ 28. Here, we tested the alteration of Rac1 activity, the relationship between Rac1 and PKCζ and the involvement of Rac1 in actin rearrangement upon PA stimulation. The activity of Rac1 (Rac1-GTP bound form) increased in presence of PA. Neither AMPK inhibitor compound C (C.C) nor AMPK stimulator AICAR impacted Rac1 activity. C.C could not block PA-induced Rac1 activation. CD36 or Fyn knockdown could activate Rac1, and PA stimulation could not further activate Rac1 (Figure 6A). Although PS (PKCζ inhibitor) blocked PA-induced actin remodeling (Figure S4), CD36 translocation, and fatty acid uptake (Figure 5), it had no effects on Rac1 activation (Figure 6A). The results indicated that Rac1 could be activated by PA, whereas CD36 and Fyn regulated Rac1 activity negatively. The relationship between Rac1 and actin was examined by immunostaining. Compared with the control group (Figure 6B, a-c), PA stimulation caused Rac1 translocation to dorsal surface of the cell and co-localization with newly formed actin remodeling (Figure 6B, d-f), which could be eliminated by HA-tagged dominant negative Rac1 (Rac1 DN) transfection (Figure 6C, a-c). Despite the absence of PA treatment, HA-tagged constitutively active Rac1 (Rac1 CA) transfection could induce actin remodeling (Figure 6C, d-f). PA/Rac1 CA-induced dynamic change of actin could be blocked by PKCζ inhibitor PS (Figure 6C, g-i). Since PS had no inhibition effect on Rac1 (Figure 6A), we might conclude that Rac1 located upstream of PKCζ. It has been ever reported that PKCζ activity was regulated by Rac-1 49. It was probable that Rac1 might participate in PA-induced actin remodeling through PKCζ in muscle cells. Before PA treatment, endogenous PKCζ and Rac-1 showed scattered distribution in the cells (Figure 6D, a-c). Co-localization of PKCζ and Rac1 were not observed. After PA treatment, both endogenous PKCζ and Rac-1 reorganized into actin mesh-like structures (Figure 6D, d-f). These data suggested a PA-mediated spatial association between PKCζ and Rac1 in myotubes. To further characterize the association of PKCζ with Rac1, differentiated L6myc cells were transiently transfected with Rac1 CA and Rac1 DN respectively. Immunostaining results revealed the relocation and co-localization of transfected Rac1 CA and endogenous PKCζ in cells (Figure 6D, g-i). The overexpression of Rac1 CA recruited PKCζ to membrane scaffold, where CD36 was compartmentalized by filamentous actin (Figure 6D, g-i and Figure S4A). In cells transfected with Rac1 DN, PA failed to induce formation of actin-remodeling-like staining of both endogenous PKCζ and Rac1 DN (Figure 6D, j-l). These data suggested that actin remodeling induced by either PA or Rac1 CA was mediated by PKCζ. At the same time, the transfection of Rac1 CA could bring ∼40% increase on FA uptake in basal state and the introduction of Rac1 DN could eliminate PA-induced FA uptake ∼75% (Figure 6E). The results indicated that Rac1-PKCζ-actin pathway was required yet insufficient in PA-induced CD36 translocation and FA uptake, and there must be other employed mechanism/pathways in this process.

### TBC1D1 was involved in Palmitate-induced CD36 translocation

**Figure 7:**
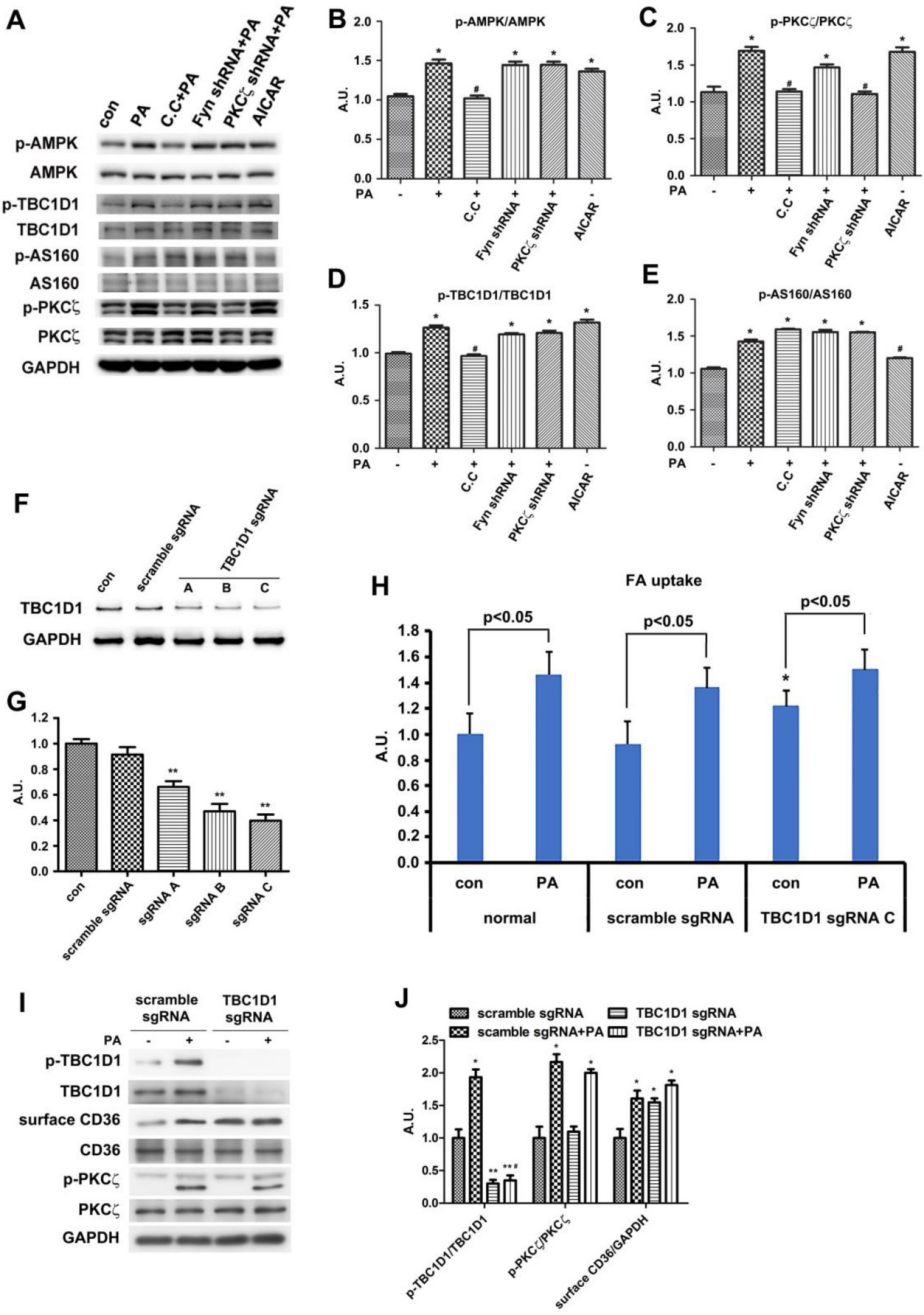
** TBC1D1 was involved in PA-induced CD36 translocation. (A) TBC1D1 was phosphorylated by AMPK.** Cells were treated with or without PA; or cells were pretreated with 20 μmol/L C.C followed by PA stimulation for another 1 h; or cells were treated with 2 mmol/L AICAR only for 1 h; or cells were transfected with Fyn shRNA and PKCζ shRNA respectively, then PA was added after 48 h and maintained for another 1 h. Whole cell lysis protein was immunoblotted with indicated antibodies. **(B), (C), (D) and (E) The quantitative analysis of western blot results.** The protein bands were quantified. Data were means ± SE (n=3), *p<0.05 vs. con, ^#^*p*<0.05 vs. PA, by t-test. **(F) Assessment of TBC1D1 sgRNAs and (G) Quantitative analysis of western blot results.** Cells were transfected with TBC1D1 sgRNAs and maintained for 48h. Whole cell lysis protein was immunoblotted with indicated antibodies. Data were means ± SE (n=3), **p<0.01 vs. scramble by t-test. **(H) Effect of TBC1D1 knockout on FA uptake.** sgRNAs was transfected into the cells and maintained for 48h. Then cells were left untreated or treated with PA for 1h. FA uptake was measured. Data were means ± SE (n=3), *p<0.05 vs. normal con by test. **(I) Effect of TBC1D1 knockout on CD36 distribution and PKCζ activity and (J) Quantitative analysis of western blot results.** Cells were transfected with sgRNAs as indicated and cells were left untreated or treated with PA for 1h after 48 h. Then proteins were detected with corresponding antibodies. Data were means ± SE (n=3), *p<0.05 and **p<0.01 vs. scramble sgRNA, ^#^*p*<0.05 vs. TBC1D1 sgRNA+PA, by t-test.

It has been reported that two Rab-GTPase activating proteins (Rab-GAP), namely TBC1D1 and TBC1D4 (AS160), were involved in regulation of both insulin- and contraction-induced glucose uptake in intact mouse skeletal muscle via modification of GLUT4 translocation 50, 51. Phosphorylation of TBC1D1 and AS160 in response to physiological stimuli in human skeletal muscle indicated that Akt and AMPK might be upstream kinases 34. We have demonstrated that the activities of AMPK and PKCζ were increased by PA stimulation, and PA had no effect on Akt. On the other hand, PKCζ-CA overexpression had no effect on the phosphorylation of TBC1D1/4. Thus, we hypothesized that TBC1D1 and/or AS160 might also regulate CD36 distribution secondary to AMPK activation. Upon PA stimulation, the phosphorylation of AS160 and TBC1D1 together with activation of AMPK and PKCζ increased (Figure 7A-E). When cells were pre-treated with AMPK specific inhibitor compound C (C.C), PA-induced activation of AMPK/PKCζ and phosphorylation of TBC1D1 decreased, yet phosphorylation of AS160 persisted. When Fyn shRNA was transfected into cells and followed up by PA treatment, the effects were similar to sole PA treatment. In the case of PKCζ shRNA transfection, although PA-induced PKCζ activity dropped, the activation of AMPK and phosphorylation of AS160 and TBC1D1 maintained. If the cells were treated with only AMPK activator AICAR, AMPK and PKCζ activity as well as TBC1D1 phosphorylation were increased. Intriguingly, AICAR had no effect on phosphorylation of AS160 (Figure 7A-E). The results revealed that phosphorylation of both AS160 and TBC1D1 increased in response to PA treatment, but only TBC1D1 phosphorylation related to AMPK activation despite of PA or AICAR induction. The above data revealed that AMPK-dependent TBC1D1 phosphorylation was required for PA-induced CD36 translocation and FA uptake. It seemed that AS160 phosphorylation was unrelated to CD36 translocation although its phosphorylation increased upon PA treatment. To further demonstrate the role of TBC1D1 in PA-induced CD36 redistribution, three TBC1D1 specific sgRNA were transfected into cells to block TBC1D1 expression (Figure 7F-G). TBC1D1 knockdown could increase FA uptake ∼50% and PA treatment could bring about more FA uptake (Figure 7H). Western blot indicated that TBC1D1 knockdown increased content of surface CD36, which mimicked the effect of PA stimulation. PA could further increase surface CD36, yet its effects were insignificant (Figure 7I-J). TBC1D1 knockdown had no effect on PKCζ activity. Since AS160 has ever been shown to regulate CD36 trafficking in cardiomyocytes 52, the role of AS160 was further examined in L6 skeletal muscle cells when AS160 was knocked down or AS160 4P (AS160 with four Akt phosphorylation sites Ser318, Ser588, Thr642, and Ser751 mutated to alanine) was overexpressed. The results showed that there was no significant increase of surface CD36 and FA uptake after AS160 knockdown. At the same time, PA stimulation further increased surface CD36 and FA uptake. The overexpression of AS160 4P did not change basal and PA-induced CD36 relocation and FA uptake significantly (Figure S5). Compared with the effect of TBC1D1 knockdown, it was obvious that TBC1D1 mainly contributed to PA-induced CD36 relocation and FA uptake in skeletal muscle cells. Collectively, it was TBC1D1, rather than AS160, that located downstream of AMPK and played the main role in mediating PA effect. Rab8a and Rab14 were shown to function in GLUT4 recycling in L6 skeletal muscle cells 53. Whether they functioned in mediating the effect of TBC1D1 on CD36 vesicles translocation still needed further research.

### Dual inhibition of AMPK and Rac1 inhibited PA effect

**Figure 8:**
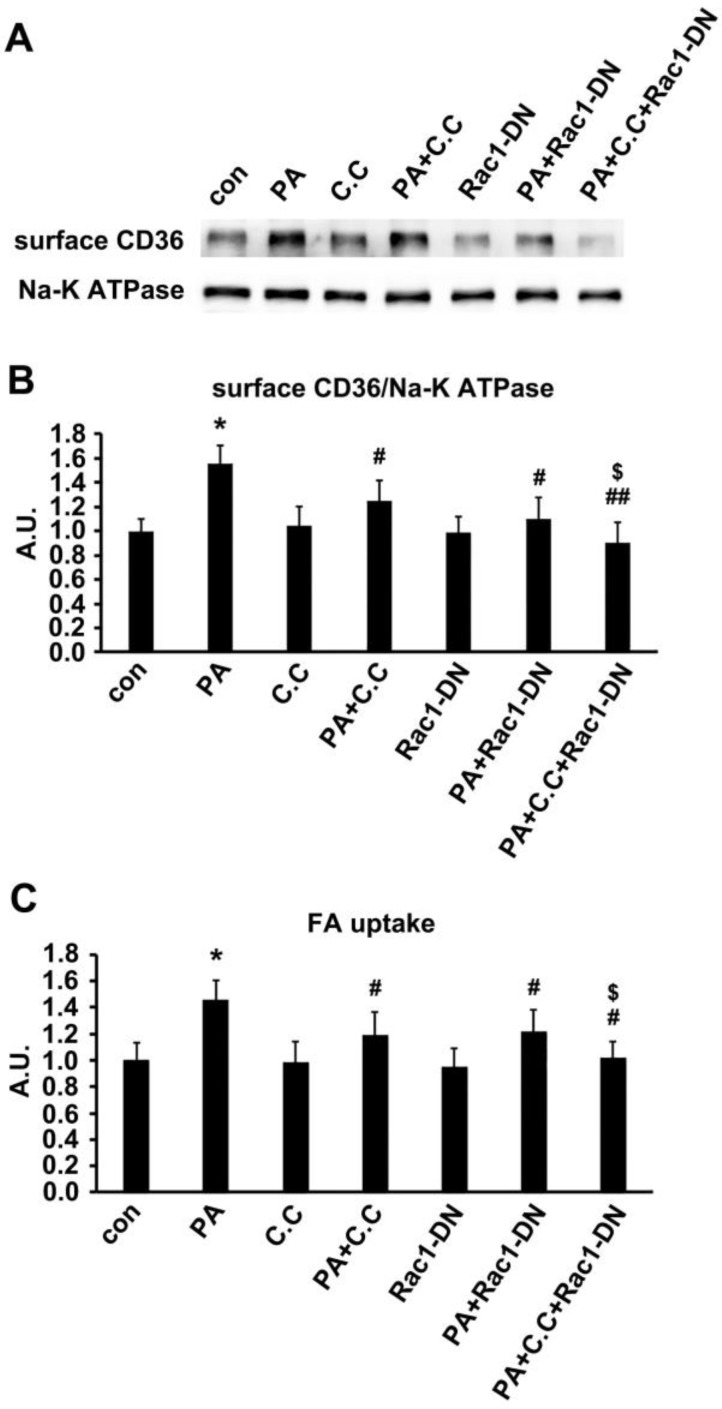
** Effect of dual inhibition of AMPK and Rac1 on PA-induced CD36 translocation and FA uptake. (A) Surface CD36 detection.** Cells were treated with or without PA; or cells were pretreated with 20 μM C.C for 20 min followed by PA stimulation for another 1 h; or cells were transfected with Rac1-DN plasmid, then PA was added after 48 h and maintained for another 1 h; or cells were transfected with Rac1-DN plasmid, then cells were pretreated with 20 μM C.C for 20 min followed by PA stimulation for another 1 h. Whole cell lysis protein was immunoblotted with indicated antibodies. **(B) The quantitative analysis of western blot results.** The protein bands in A were quantified. A value of 1 was assigned to the control condition. Data are means ± SE (n=3), *p<0.05 vs. con, ^#^*p*<0.05 or ^##^*p*<0.01 vs. PA, ^$^*p*<0.05 vs. PA+C.C/PA+Rac1-DN, by t-test. **(C) FA uptake assay.** Cells were treated with or without PA; or cells were pretreated with 20 μM C.C for 20 min followed by PA stimulation for another 1 h; or cells were transfected with Rac1-DN plasmid, then PA was added after 48 h and maintained for another 1 h; or cells were transfected with Rac1-DN plasmid, then cells were pretreated with 20 μM C.C for 20 min followed by PA stimulation for another 1 h. FA uptake was measured. Data were means ± SE (n=3), *p<0.01 vs. con by t-test. *p<0.05 vs. con, ^#^*p*<0.05 vs. PA, ^$^*p*<0.05 vs. PA+C.C/PA+Rac1-DN, by t-test.

Since TBC1D1 phosphorylation and PKCζ activation independently contributed to PA-induced CD36 translocation. We next explored the effect of dual inhibition on the two pathways. Given the evidence that TBC1D1 knockdown could increase surface CD36 (Figure 7) whereas PKCζ knockdown blocked PA-induced CD36 relocation (Figure 5), it was better to simultaneously inhibit AMPK and Rac1, the two kinases that locate upstream of TBC1D1 and PKCζ, to examine the effect of dual inhibition of the two pathways on PA-induced FA uptake. Cells were first transfected with Rac1-DN, followed by the treatment of compound C (C.C, AMPK inhibitor). The results showed that either Rac1-DN or C.C could partially inhibit PA-induced CD36 relocation (Figure 8A-B) and FA uptake (Figure 8C), and their combination could completely block PA-induced CD36 translocation and FA uptake with an additive effect.

### Oleic acid showed similar effect to palmitate

**Figure 9:**
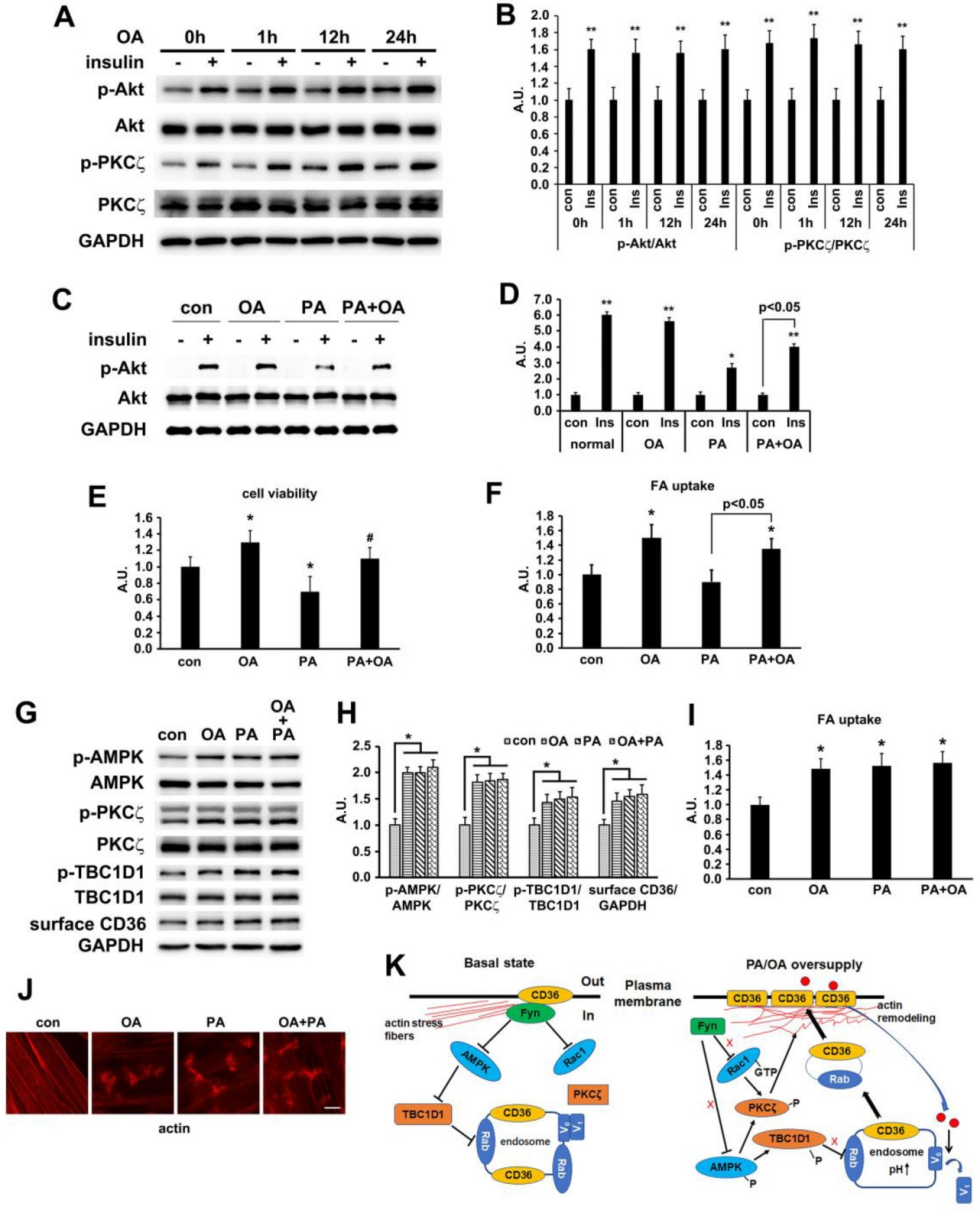
** Oleic acid showed similar effect as palmitate. (A) Effect of OA on insulin sensitivity.** Cells were left untreated or treated with 500 μmol/L OA for different time followed by adding insulin and incubation for another 10 min. The total protein was extracted and immunoblotted with indicated antibodies. **(C) OA protected PA-induced insulin resistance.** Cells were left untreated or treated with OA, PA or PA+OA for 24h followed by adding insulin and incubation for another 10 min. The total protein was extracted and immunoblotted with indicated antibodies. **(G) Effect of OA on kinases activation and CD36 distribution.** Cells were left untreated or treated with OA, PA and OA+PA for 1h respectively, then whole cell lysis protein was immunoblotted with indicated antibodies. **(B), (D) and (H) The quantitative analysis of western blot results.** The protein bands in A, C and G were quantified. A value of 1 was assigned to the control condition. Data are means ± SE (n=3), *p<0.05 and **p<0.01 vs. con by t-test. **(E) OA improved the decrease of cell viability induced by PA.** Cells were left untreated or treated with OA, PA or PA+OA for 24h, then cell viability was measured by MTT method according to the kit instruction. *p<0.05 vs. con, ^#^*p*<0.05 vs. PA, by t-test. **(F) and (I) FA uptake assay.** Cells were left untreated or treated with OA, PA and OA+PA for 24h (F) or for 1h (I) respectively, then FA uptake was measured. Data were means ± SE (n=3), *p<0.01 vs. con by t-test. **(J) Effect of OA on actin remodeling.** Cells were left untreated or treated with OA, PA and OA+PA for 1h respectively, then cells were fixed and stained for actin (red), Bar, 10 μm. The images are representative of three experiments. **(K) Schematic signal transduction induced by FA.** Upon fatty acid oversupply, resident CD36 molecules on the membrane may initiate signal transduction leading to disassociation of Fyn from surface CD36, which in turn activates AMPK and Rac1 respectively. AMPK locates upstream of PKCζ and controls the activity of PKCζ whereas Rac1 facilitates the translocation of PKCζ to the dorsal surface of the cell to cause actin remodeling. Furthermore, AMPK can also phosphorylate TBC1D1 to release the retained cytosolic CD36 to facilitate FA uptake. The FA transported into the cells increases endosomal pH via v-ATPase disassembly, which further promotes CD36 vesicles relocation under the condition of TBC1D1 phosphorylation and PKCζ activation. Thus, the dual modulation of PKCζ and TBC1D1 finally results in a regulation of sarcolemmal CD36 translocation through positive feedback.

In our *in vivo* experiment, the main difference between HFD and NCD was the lard addition that accounted for 20% of energy uptake. It has been well documented that palmitate (PA, 16:0) and oleic acid (OA, 18:1) were the two main components of lard 54, 55. We first evaluated the effect of OA on insulin-induced activation of PKCζ/Akt at different time points. Compared with PA (Figure 2C), OA did not interfere with insulin sensitivity, suggesting that insulin sensitivity maintained after OA treatment for at least 24h (Figure 9A-B). Furthermore, the effects of PA, OA and PA+OA on cell viability (Figure 9C), insulin sensitivity (Akt phosphorylation, Figure 9D) and fatty acid uptake (Figure 9E) were examined during cell exposure to fatty acid for a long time (24 h). The lipotoxicity induced by long-term PA treatment could be ameliorated by OA. Following that, we wanted to know whether OA could induce CD36 translocation in the same mechanism as PA. When L6 myotubes were treated with OA and PA for a short time (1 h) respectively, the activation of AMPK/PKCζ, actin remodeling, phosphorylation of TBC1D1, surface CD36, and fatty acid uptake were increased. Combination of OA and PA did not perform a synergistic effect (Figure 9 G-J). The results indicated that the signal transduction induced by OA was similar to PA and thus the signaling mechanism was universal to fatty acid uptake (not only specific to PA). Although both PA and OA could lead to CD36 relocation in the same manner and cause similar fatty acid uptake, there was no synergistic effect, suggesting a possible competition between PA and OA in binding sarcolemmal CD36 to promote FA transport. The probable reason for OA to reverse lipotoxocity of PA was that the metabolic fates of OA was different from PA after fatty acids uptake, which in turn caused opposite effect on insulin sensitivity and cell viability. Indeed, when excess PA was transported into the cells, it increased the synthesis of deleterious lipids, such as diacylglycerol (DAG) and ceramide, which in turn attenuated the insulin signaling pathway and increased lipotoxicity 10, 11. OA transported into cells could reduce the synthesis of DAG and ceramide by promoting mitochondrial oxidation of PA and accumulation of PA in the form of triacylglycerol (TAG) 56.

## Discussion

Fatty acid oversupply might cause lipid overconsumption and thus accumulation of triglyceride, diacylglycerols and ceramides in skeletal muscle cells. The molecular mechanisms of these lipid metabolites inducing insulin resistance and type 2 diabetes have been well studied. However, it was uncertain how CD36 translocates to plasma membrane for mediation of FA uptake upon FA being the dominant energy source and prior to insulin resistance. Here, we showed that cell surface CD36 molecules might bind with fatty acid extracellularly and initiate a signal transduction that caused more CD36 translocation from cytosol to plasma membrane. The elevated translocation transported more lipid into the cells before the onset of insulin resistance. Experimental results might help us understand the initial events leading to CD36 translocation and fatty acid uptake in response to FA oversupply. The results also revealed the molecular mechanism of CD36 relocation in high fat feeding rodent models 23, 24. The results obtained could help us understand the initial events leading to CD36 translocation and FA uptake in response to FA oversupply.

In this study, HFD-fed mice (Figure 1) and PA-treated myotubes (Figure 2) for different timeframes could dissect the process of insulin resistance, which made it possible for us to explore the molecular mechanism that led to lipid overconsumption due to FA oversupply when insulin signal transduction remained normal. Our experimental results confirmed the time course of events during development of high fat-induced insulin resistance in rodents 23, 24 and palmitate-induced insulin resistance in cardiac myocytes 57. Studies in rodents 23, 24 have shown that short-term exposure to HFD resulted in rapid development of insulin resistance and glucose intolerance. Here, we showed that insulin sensitivity deteriorated after two weeks of HFD-feeding in mice. Notably, lipid accumulation in muscle occurred (1wk) before the elevation of plasma insulin and onset of insulin resistance (Figure 1), implying the importance of lipid species in the induction of insulin resistance 7. Prior to the metabolic change of lipids (3d), we found that the increase of surface CD36 was accompanied by elevated AMPK/PKCζ activity. The data indicated that CD36 redistribution on plasma membrane was induced by FA rather than insulin. Surface CD36 was compulsory for excess FA uptake, which might lead to ectopic accumulation of intramyocellular lipids and impairment of insulin function. Similarly, both surface CD36 and AMPK/PKCζ activity increased before the impairment of insulin signal transduction when myotubes were treated with palmitate (Figure 2). Both *in vivo* (Figure 1) and *in vitro* (Figure 2) studies showed that CD36 and GLUT4 molecules were present on the plasma membrane in basal state to faciliatate fatty acid and glucose uptake. Insulin sensitivity persisted in three-day HFD-fed mice or one hour PA incubated myotubes. At this stage, there was significant increase of surface CD36 yet no surface GLUT4 alterations. The results indicated that FA supply could stimulate CD36 translocation to plasma membrane without affecting GLUT4 distribution. Consistently, it has been reported that CD36 inhibition prevented lipid accumulation in cells 57.

Our results clearly showed that PA-induced AMPK/PKCζ activation, CD36 translocation, and FA uptake were inhibited by SSO (Figure 3). The hydrophobic pocket binding site of CD36 for SSO and FA were similar 41 and thus SSO functioned as an antagonist of FA. Even though PA and SSO shared similar binding sites on CD36 molecules, PA could initiate signal transduction via binding surface CD36 whereas SSO could not. Therefore, the signal transduction was most likely modulated by up taken palmitate. It was also possible that binding of PA with CD36 caused a CD36 conformational change different from that of SSO-induced. This structural change of CD36 led to the disassociation of Fyn from CD36 in cytosol.

It has been reported that CD36-Fyn complex could control the distribution of LKB1 to regulate AMPK activity 46. Furthermore, Fyn null mice and SU6656 treated wild type mice displayed activation of AMPK and increased FA oxidation 58. Upon Fyn knockdown by shRNA or inhibition by SU6656, LKB1 translocated from nucleus to cytosol while AMPK activity, surface CD36, and FA uptake increased (Figure S1). The results indicated that Fyn played a negative role in controlling AMPK activation, CD36 sarcolemma translocation, and FA uptake in response to FA supply. Intriguingly, both AMPK and PKCζ activation increased when CD36 was knocked down (Figure 3), suggesting the native inhibition effect of CD36 on AMPK and PKCζ. Thus, CD36 knockout could reduce its association with Fyn, leading to cytosolic enrichment of LKB1 and phosphorylation of AMPK, which also supported Samovski's work 46. On the other hand, Rac1 was activated when CD36 or Fyn was inhibited, implying that CD36-Fyn complex not only regulated AMPK activity but also impacted Rac1 activation (Figure 6). PKCζ located downstream and was the convergent of these two kinases.

It has been well established that aPKCs-Par6-Par3 complex controlled cell polarity via regulating actin structure 59. We have ever reported that Rac1 influenced PKCζ activity and PKCζ further mediated insulin-induced GLUT4 translocation through actin 28. F-actin was required for insulin stimulated GLUT4 translocation and glucose uptake 60, 61. It was also plausible that Rac1-aPKCs could mediate FA-induced CD36 translocation via regulating actin structure. Indeed, actin destruction by CB totally inhibited PA-induced FA uptake (Figure S7). Actin morphology in tissue samples showed that two-month HFD feeding could cause actin distortion along with CD36 sarcolemmal translocation, suggesting involvement of actin in CD36 vesicular movement. It should be noted that the dynamic change of actin morphology was distinct in tissue and cell line. More *in vivo* research work needs to be done to investigate the biological function of actin in mediating signal transduction and substrate transportation. In cardiomyocytes, actin was shown only to be required for insulin/oligomycin-induced glucose uptake, but not for FA uptake 62, suggesting different roles of actin in fatty acid uptake in distinct cell types. Indeed, while microtubule participated in insulin-induced GLUT4 translocation and glucose uptake in L6 skeletal muscle cells 40, it had no function in cardiomyocytes 62. Thus, roles of cytoskeleton in mediating GLUT4/CD36 vesicles transportation varied in different kinds of cells. The close association between PKCζ and Rac1 was further supported by 1) PKCζ staining pattern changes resembling new actin structures in Rac1-CA-transfected L6myc cells without FA treatment; and 2) prevention of actin reorganization and hence PKCζ re-localization by Rac1 DN transfection despite of FA stimulation (Figure 6).

Rac1 regulated the activity of PKCζ through interactions with adapter protein Par6 49, 63. These results supported the notion that aPKCs induced actin reorganization through a Rho-dependent pathway, and PKCζ functioned downstream of Rac1 64, 65. Results from Figure 6 revealed that Rac1-PKCζ induced actin remodeling was necessary yet insufficient in FA-induced signaling pathway due to inactivity of AMPK-TBC1D1 pathway. Hence, sole actin disruption could not significantly affect oligomycin (AMPK activator)-induced CD36 movement 62. PKCζ-CA overexpression did not affect AMPK activation and TBC1D1 phosphorylation (Figure 5) while TBC1D1 knockdown did not influence PKCζ activity (Figure 7). Summing up, PKCζ and TBC1D1 were two independent molecules bearing respectable functions in PA-induced signal transduction. Since PA-induced Rac1 activity was AMPK-independent (Figure 6), it was possible that AMPK and Rac1 were parallel signaling molecules branched from Fyn upon FA stimulation. AMPK then regulated CD36 distribution by mediating the PKCζ activity and TBC1D1 phosphorylation. Rac1 regulated actin remodeling through PKCζ reorganization on the dorsal surface of the cells. Thus, PKCζ was regulated by AMPK and Rac1 coordinately and participated in FA-induced CD36 transportation through actin remodeling. Our conclusion was consistent with the notion that PKCζ was required for cardiac CD36 translocation and FA uptake 66. It has ever been reported that palmitoylation was critical for targeting CD36 to the plasma membrane 67. It was probable that palmitoylated CD36 could be sorted into vesicles for TBC1D1 regulated migration into plasma membrane. PKCζ-caused actin remodeling provided a docking site (lipid raft area containing DHHC5) for these vesicles to fuse with plasma membrane and induce CD36 insertion into the membrane to mediate FA uptake. Given the evidence that PKCζ could be activated by insulin 28 and PA in this study, and only PKCζ blocking could attenuate the effect of PA and insulin -induced FA uptake (Figure S2), it was probable that PKCζ was the potential cross talk between PA and insulin -induced CD36 translocation.

Insulin promoted CD36 and GLUT4 translocation to increase FA and glucose uptake respectively 15, suggesting the translocation of these two transporters shared identical molecular mechanisms in presence of insulin. On the other hand, surface CD36 but not GLUT4 elevated when FA was dominant energy source 68, implying differences between their translocations. Indeed, Liu et al. has ever reported that palmitate exposure could inhibit v-ATPase function to promote CD36 sarcolemmal translocation without affecting GLUT4 distribution 69. Phosphorylation states of TCB1D1 and AS160, which controlled vesicular trafficking via regulating the activity of Rabs, might account for the differences in CD36 and GLUT4 distribution. Thus, we examined the phosphorylation of both TBC1D1 and AS160 upon FA stimulation. Although the phosphorylation of these two RAPs increased after PA treatment, only TBC1D1 phosphorylation was mediated by AMPK (Figure 7). As AMPK inhibition blocked FA-induced CD36 translocation and FA uptake significantly (Figure 5 and Figure 7), we concluded that TBC1D1 but not AS160 was mainly involved in CD36 movement. TBC1D1 knockdown experiments further proved the conclusion (Figure 7). AMPK and Akt were upstream kinases of TBC1D1 and AS160 34, yet there was no change in Akt activity after FA oversupply (Figure 1-3). Thus, there must be other upstream kinases apart from AMPK and Akt that phosphorylated AS160. Even though AS160 phosphorylation increased in response to FA stimulation, it did not contribute to CD36 translocation significantly. The role of AS160 in this study was inconsistent with Samovski's report 52 and this discrepancy might be due to the distinct cell models from different tissue sources. A limitation of the study was that one functional parameter - muscle contraction was not measured. Due to the model systems chosen in this project not allowing such measurements easily, the effects of PA on CD36 relocation via dual modulation of PKCζ and TBC1D1 were tested and confirmed in C2C12 skeletal muscle cells, which could also be induced into insulin resistance 70. The experimental results obtained from C2C12 cells were consistent with that of L6 cells (Figure S6).

In summary, HFD fed mice and palmitate-treated L6 skeletal muscle cells were used to explore the underlying molecular mechanisms of CD36 translocation to plasma membrane prior to insulin resistance. As shown in Figure 9K, resident CD36 molecules on the membrane would initiate a signal transduction upon FA oversupply, leading to PKCζ activation and TBC1D1 phosphorylation, hence formulating positive feedback regulation of CD36 sarcolemmal translocation to mediate FA uptake. Identified targets could be adopted in precision medicine to potentially treat lipid oversupply-induced insulin resistance.

## Materials and Methods

### Antibodies and Chemical reagents

Tissue culture medium, serum, and other tissue culture regents were purchased from Invitrogen (Carlsbad, CA). Soluble insulin was purchased from Novo Nordisk (Bagsværd, Denmark). ProLong Antifade mounting solution, 4, 6-diamidino-2-phenylindole (DAPI), phalloidin, and Alexa Fluor anti-rabbit or anti-mouse secondary antibodies were purchased from Invitrogen. Antibody against Thr410/403 PKCζ/λ, Ser473 Akt and Akt substrate, Thr172 AMPK, Ser700 TBC1D1 were purchased from Cell Signaling Technology (Beverly, MA). Antibody against TBC1D1 was purchased from Thermofisher (Waltham, MA). Polyclonal antibody against PKCζ/λ (H1), goat anti-rabbit, and goat anti-mouse secondary antibodies were purchased from Santa Cruz Biotechnology (Santa Cruz, CA). Mono- and poly-clonal antibodies against CD36 were purchased from Abnova (Taipei, TW). Bio-Rad protein assay was purchased from Bio-Rad (Hercules, CA). Enhanced chemical luminescence (ECL) [γ-^32^P]-ATP, 2-deoxy-[^3^H]-D-glucose, and protein G beads were purchased from GE Healthcare (Little Chalfont, Buckinghamshire, United Kingdom). Antibody against Rac-1, [^159^Ser] PKC-ε (AA153-164)-NH_2_ were purchased from Upstate Biotechnology (Lake Placid, NY). Phenylmethylsulfonyl fluoride (PMSF) was purchased from Calbiochem (San Diego, CA). Cell-permeable PKCζ pseudosubstrate (PS) myristoyl trifluoroacetat and other necessary chemical reagents were obtained from Sigma-Aldrich (St. Louis, MO). Lipofectmine 3000, Opiti-MEM, and pEGFP-N1 vector, Fluorescently labeled deoxyglucose 2-NBDG (2-[*N*-(7-nitrobenz-2-oxa-1,3-diazol-4-yl)amino]-2-deoxy-D-glucose),BODIPY™500/510C_1_,C_12_ (4,4-Difluoro-5-Methyl-4-Bora-3a,4a-Diaza-*s*-Indacene-3-Dodecanoic Acid) were purchased from Invitrogen (Waltham, MA). G418 was purchased from Alexis Biochemicals (Lausen, Switzerland). HA-tagged constitutively active (CA) and dominant negative (DN) Rac1 were purchased from cDNA Recourse Center (Bloomsburg, PA).

### Experimental animals

Male C57BL/6 mice (four weeks old) were purchased from Guangzhou Animal Center, China. Mice were housed in a temperature-regulated environment (20°C) with a reversed 12:12 hour light-dark cycle. The principles of laboratory animal care were followed and the experiments were approved by the Animal Care Committee, Shenzhen University. After one week of adaptive feeding, mice were divided randomly into normal-chow-diet (NCD) and high-fat-diet (HFD) groups. NCD contained (wt/wt) 4.3% fat, 19.2% protein, and 67.3% carbohydrate. HFD contained (wt/wt) 24% fat, 24% protein, and 41% carbohydrate. Both diets were otherwise identical and manufactured by Jiangsu Medicience Ltd, China. Eight to ten mice of each group were sacrificed at 3d, 1wk, 2wk and 4wk respectively, and body weight was monitored before sacrificing. After intraperitoneal injection of pentobarbital (1%, 50mg**/**kg), blood samples and hind limb muscles (soleus muscle, extensor digitorum longus muscle and tibialis anterior muscle) were collected. Hind limb muscles were used for giant sarcolemmal vesicles isolation immediately or fixed by 4% paraformaldehyde followed by immunostaining. Blood was immediately centrifuged, and the plasma was separated and stored at -80°C until assayed for blood glucose and lipid profile.

### Insulin tolerance test (ITT) and oral glucose tolerance test (OGTT)

The dynamic characteristics of blood glucose after short insulin injection was assessed by ITT. All animals were fasted for 6 h. Ultra-short action insulin was administered into abdominal cavity (Actarapid Nova Nordisk, Bangalore; Dose: 0.5 IU/ kg; ip). Blood was collected by tail cut, and glucose levels were measured by glucometer at 0, 15, 30, 60 and 120 min after insulin injection. OGTT was conducted after 6 hour's fasting. Glucose (2g/kg) was delivered into the stomach by a gavage needle (20-gauge, 38 mm long curved, with a 21/4 mm ball end). 10μl of blood was sampled at 0, 30, 60, 90 and 120 min for plasma glucose. The OGTT was performed one day before the sacrifice.

### Plasma analysis

Plasma insulin was measured by ELISA (Millipore, St Louis, MO, USA). Plasma free fatty acid was measured by Biochemical analyzer.

### Lipid analysis

TG and DAG in skeletal muscle tissue homogenates were measured by ELISA (MBS776029, MBS777065, San Diego, CA, USA).

### L6 cells culture and experiments

L6 muscle cells expressing c-myc epitope tagged GLUT4 (L6myc cells) 71, 72 were maintained in myoblast monolayer culture in α-minimal essential medium containing 10% (vol/vol) fetal bovine serum ( FBS) and 1% (vol/vol) antibiotic-antimyocotic solution (100 U/ml penicillin G, 10 μg/ml streptomycin, and 25 mg/ml amphotericin B) in an atmosphere of 5% CO2 at 37°C. For differentiation into myotubes, myoblasts were plated in medium containing 2% (vol/vol) FBS at 10^4^ cells/ml to allow spontaneous myoblast fusion. Medium was replaced every 48 h, and myotubes were ready for experiment 6-8 d after plating. Transfection of L6 GLUT4myc myotubes was performed in six-well plates as described previously 28. In brief, DNA was introduced into the cells at the start of day 4, and cells were maintained for another 48 h until experimentation. For stable transfection, 2 μg of DNA was introduced into the myoblasts at 10^4^ cells/ml, and cells were maintained for another 48 h before incubation in normal culture medium containing 0.5 mg/ml G418. Proper clones were selected by immunofluorescence and subcultured with G418 throughout the entire course. The transfected cells were used within six generations.

### Glucose and fatty acid uptake assay

Cells were prepared using 24-well plate with black wall and clear bottom. After pretreatment with drugs, the supernatant was aspirated and cells were washed with pre-warmed PBS twice. 2-NBDG based glucose uptake was measured according to the reported methods 73, 74 with modification. The working concentration for 2-NBDG was 0.1 mmol/L in HEPES containing 0.2% fatty acid-free BSA. For fatty acid uptake assay, BODIPY 500/510 C1, C12 in HEPES containing 0.2% fatty acid-free BSA (the working concentration for BODIPY was 0.1 mmol/L) was added into the plate and incubated for 30 min at 37ºC. We set two kinds of blank for FA uptake: 1) The cells in blank wells were incubated with BODIPY-FA solution containing 10 μmol/L Cytochalasin B (CB) to significantly destroy actin structure, inhibiting all life activities including fatty acid transport; 2) Cells were incubated with 500 μmol/L PA+100 μmol/L BODIPY-FA. Then the unlabeled PA competed for the uptake of BODIPY-FA (Figure S7). The cells were washed with pre-warmed PBS twice. Finally, 500 ul of PBS was added into each well. The fluorescence signal was measured with Cytation-3 multi-mode reader (Bio-Tek) at Ex/Em=488/515 nm using a bottom read mode. In order to correct possible adherent contamination of BODIPY-FA on the membrane, mean of the two blank values was excluded from control and experimental group value. The plates were then taken out. Upon aspiration of PBS, 100 µl cold RIPA (50 mmol/L Tris-HCL pH 7.4, 1% NP-40, 0.5% Na-deoxycholate, 0.1% SDS, 150 mmol/L NaCl, 2 mmol/L EDTA) was added into each well to lyse the cells on ice for 30 min. The protein concentration was measured to calculate the total protein content. The fluorescence value was modified by total protein content. Each condition was assayed with triplicate.

### Surface CD36 and GLUT4 detection

Cells were plated on 10 cm culture dish for surface protein isolation. Cells were washed three times with cold PBS followed by a 60-minute incubation with freshly prepared EZ-Link Sulfo-NHS-SS-Biotin (Pierce, Rockford, IL, USA; 1 mg/ml in PBS) on ice. Then, non-reacted biotin was neutralized with 100 mmol/L cold glycine for 15 minutes, and cells were washed twice with cold PBS. 500 µl lysis buffer (50 mmol/L Tris-HCl pH7.4, 2% Triton X-100, 150mmol/L NaCl, 1mmol/L EDTA, 0.1% SDS, 10% glycerol, 1mmol/L PMSF, 1mmol/L NaF, 1% proteases inhibitor cocktail (Sigma-Aldrich, St. Louis, MO)) was added to the cells. Cells were scraped off the plates and transferred to an Eppendorf tube followed by incubation on ice for 30 min. The cell extract was centrifuged at 12000 rpm for 10 min at 4ºC. The protein concentration of the supernatant was determined using the BCA (Thermo Fisher Scientific, Pierce Rockford, IL, USA) protein assays. Equal amounts of protein (~1 mg) from each extract were used for cell surface protein isolation. The streptavidin beads were washed by PBS and balanced with cell lysis buffer. Each cell extract was mixed with 30 µl bed volume beads in one Eppendorf tube and cell lysis buffer was added to maintain a total volume of 500 µl. The Eppendorf tubes were shaking overnight. The supernatant was aspirated and the beads were washed four times with cold cell lysis buffer. The washing buffer was removed completely followed by adding 80 µl 1 X SDS loading buffer to vibrate the beads thoroughly. The samples were heated at 60ºC with shaking and centrifuged at 12000 rpm for 5 min at room temperature. Then, 20 μl supernatant was analyzed by western blot each time.

### Western blot

Aliquots proteins were separated by SDS-PAGE (10% polyacrylamide). Thereafter, proteins were electrophoretically transferred to polyvinylidene difluoride membrane and blocked in 5% BSA and 0.05% Tween 20 in Tris-buffered saline (TBST) for 1 h at room temperature. Membranes were incubated overnight at 4°C with the indicated first antibodies. Membranes were washed (3 times for 5 min each) in TBST and incubated with horseradish peroxidase-conjugated IgG for 1 h at room temperature, followed by additional washes (3 times for 15 min) in TBST. Proteins were visualized by ECL and quantified by densitometric scanning with Image J and analyzed with GraphPad Prism software. Pixel density of phosphorylation band was corrected after dividing by the corresponding protein level and showed as fold of non-treatment control; a value of 1 was assigned to the control condition.

### Immunostaining and signal detection

Skeletal muscle was excised freshly and fixed in 4% paraformaldehyde for 24 h. The tissues were dehydrated with sucrose and then were embedded in OCT solution at -80°C. The tissues were sliced to 5μm thickness for staining. For cells, after proper treatments, myotubes were fixed with 3% (vol/vol) paraformaldehyde in PBS overnight at 4°C and then washed with 0.1 mol/l glycine in PBS for 10 min. Both tissue and cell samples were permeabilized with 0.1% (vol/vol) Triton X-100 in PBS for 3 min, and then blocked with 5% BSA. After washing with PBS, the samples were incubated with primary antibodies and subsequently incubated with either Alexa-conjugated goat anti-rabbit or anti-mouse secondary antibodies respectively. For staining actin, samples were incubated with Alexa-labeled phalloidin. Samples were mounted in ProLong Antifade solution containing DAPI onto glass slides and were examined with immuno-microscope (Carl Zeiss, Jena, Germany). The content of cytosolic and nuclear protein was quantified using the Image J software (National Institutes of Health). Images of 30 representative cells were examined and results represent mean ± SE from 3 independent experiments.

### Transfection of sh/sgRNAs

All the shRNAs in this work were obtained from Origene (Rockville, MD). All the commercial plasmids contained GFP gene that had been linked with shRNA expression cassette, which made the GFP protein be co-expressed as a marker with shRNA to label transfected cells. After testing, the shRNA sequence of CD36 was 5'-GGACCATTGGTGATGAGAAGGCAAACATG-3'; the shRNA sequence of Fyn was 5'-GATAAAGAAGCAGCGAAACTGACGGAGGA-3'; the shRNA sequence of PKCζ was 5'-AGCCTCCAGTAGATGACAAGAACGATGGT-3'; the sgRNA sequence of TBC1D1 was 5'-CTCGATGCACTCGTCGATCA-3'. The sh/sgRNAs were transfected into the cells at the start of day 4 of differentiation with X-treme GENE HP DNA Transfection Reagent (Roche, St. Louis, MO) according to the instructions of the kit. Then cells were maintained for another 48 h to express the exogenous genes.

### Kinase activity assay

L6myc myotubes were left untreated or treated with 500 μmol/L PA for 1 h at 37°C. Cells were washed three times with ice-cold PBS and collected. For skeletal muscle tissues, protein was extract by grinding and then lysis with RIPA buffer. The PKCζ/λ or PKCζ enzyme activity was measured in specific immunoprecipitates as described previously by using [γ-^32^P] ATP 28. Rac1 activities were measured in the supernatant using a commercially available Rac1 activation assay kit (BK 126; Cytoskeleton Inc., CO, USA). In brief, immediately after centrifugation, the protein concentration in the lysates were determined and an equal amount of protein was loaded onto wells coated with the p-21-binding domain of PAK, which only binds Rac1 in its GTP-bound form. The amount of GTP-bound Rac1 was detected using a colorimetric assay. Fyn activity was measured by immunoprecipitation with Fyn antibody followed by the instruction of Tyrosine Kinase Activity Assay Kit (Merck, Temecula, CA).

### Statistics

Data are expressed in mean ± SEM. For Western blot, the results were quantified in the linear range by densitometry using Image J software. Differences between two means were analyzed by Student's *t* test. A two-tailed *p* value < 0.05 was considered to be significant.

## Supplementary Material

Supplementary materials and methods, figures.

## Abbreviations

aPKCs: atypical protein kinase Cs
C.C: compound C
CD: cytochalasin D
CD36: fatty acid translocase
ECL: Enhanced chemical luminescence
FA: fatty acid
FABP: fatty acid-binding protein
FATP: fatty acid transport proteins
GAPs: Rab-GTPase activating proteins
GLUT4: glucose transporter 4
HFD: high-fat diet
IR: insulin resistance
ITT: Insulin tolerance test
LCFA: long-chain fatty acid
NCD: normal-chow diet
NHS: sulfo-N-hydroxysuccinimidyl
OA: oleic acid
PA: palmitate
OGTT: oral glucose tolerance test
PI-3K: phosphoinositide 3-kinase
PMSF: Phenylmethylsulfonyl fluoride
PS: pseudosubstrate
Rac1 CA: HA-tagged constitutively active Rac1
Rac1 DN: HA-tagged dominant negative Rac1
T2D: type 2 diabetes
TBST: Tris-buffered saline
