# Supplementary material for: Lipid oversupply induces CD36 sarcolemmal translocation via dual modulation of PKCζ and TBC1D1: an early event prior to insulin resistance
Source: Theranostics. 2020 Jan 1;10(3):1332–54. doi: 10.7150/thno.40021 (PMC6956797; doi:10.7150/thno.40021)
Supplement: Supplementary file 1 — Supplementary materials and methods, figures. [file thnov10p1332s1.pdf]

Supplementary Materials

Materials and Methods

Ex vivo muscle insulin sensitivity test

Hind limb muscles of the mice were dissected and divided into two parts. Each part was preincubated for 30 min in 3 mL of modified Krebs Ringer buffer (120 mM NaCl, 4.8 mM KCl, 25 mM NaHCO3, 2.5 mM CaCl2, 1.24 mM NaH2PO4, 1.25 mM MgSO4, 8 mM D-Glucose, 2 mM sodium pyruvate, 2 mM HEPES, pH 7.4) under 95% O2 and 5% CO2 at 37°C with stirring. One of the two parts was stimulated with 100 nM insulin for 30 minutes, muscle protein was then extracted immediately for insulin sensitivity analysis.

Muscle protein extraction

Whole muscle protein was extracted by glass on glass homogenization in buffer under a ratio of 10 µl/mg of muscle. Buffer solution consisted of 50 mM HEPES (pH 7.4), 0.1% Triton-X 100, 4 mM EGTA, 10 mM EDTA, 15 mM $Na_4P_2O_7$, 100 mM β-glycerophosphate, 25 mM NaF, 5 mM Na_3VO_4, and 10 µl/ml protease inhibitor cocktail (cat. no. P8340, Sigma-Aldrich). Muscle extract was centrifuged for 10 min at 10,000 *g*, and the supernatant fraction was quantified using the BCA protein assays.

Cytosolic and nuclear protein preparation

After pre-treatment, $5x10^7$-$1x10^8$ cells were washed with cold PBS followed by adding 200ul ice cold buffer A (10mmol/L HEPES pH7.9, $MgCl_2$ 1.5mmol/L, KCl 10mol/L,

DTT 0.5mol/L, PMSF 0.5mmol/L) and pipetting gently. The cells were transferred to a 1.5ml Eppendorf tube and put on ice for 15min, then 30ul of 10% Triton X-100 was added followed by vortex for 10sec and spin at 10000rpm for 30sec at 4°C. The supernatant was removed to a new tube as cytosol protein. The pellet was re-suspended in 50ul buffer B (5mmol/L HEPES pH7.9, $MgCl_2$ 1.5mmol/L, EDTA 0.2mmol/L, DTT 0.5mol/L, glycerol 26% v/v, PMSF 0.5mmol/L), agitated vigorously and kept on ice for another 15min followed by spin at 13000rpm for 5min at 4°C. The supernatant was nuclear protein. Measure protein concentration and prepare for WB detection.

Supplemental Figures and Figure Legends

Figure S1

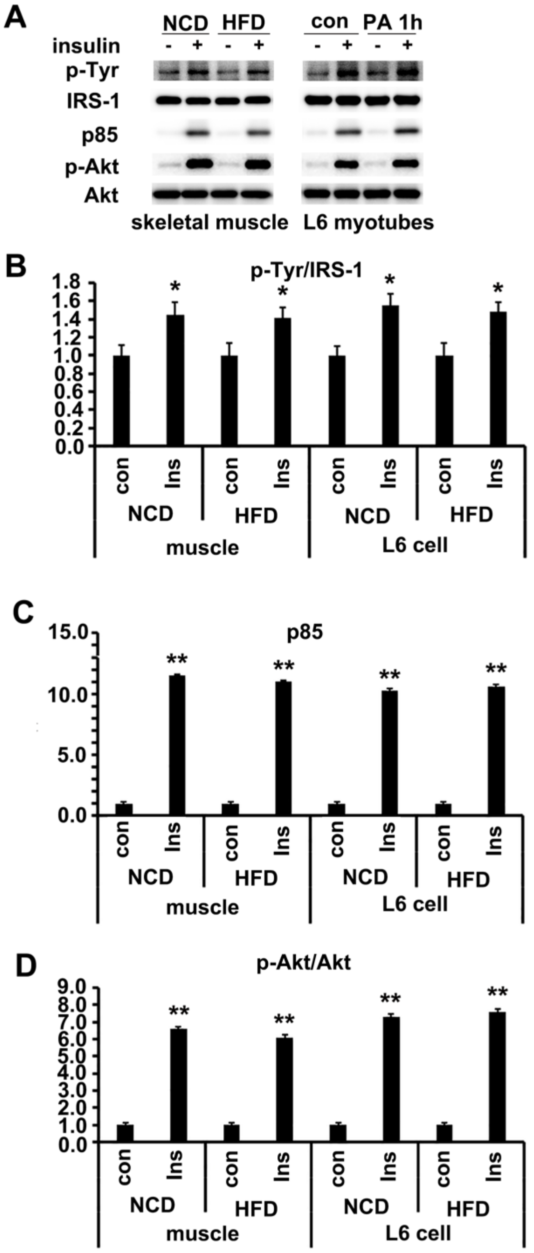

Figure S1. Insulin sensitivity detection in skeletal muscle and L6 myotubes. (A) Detection of kinases activation. After three days of feeding, skeletal muscle protein was extracted from mice in NCD and HFD groups. For L6 myotubes, the cells were left untreated or treated with 500 μmol/L PA (mol ratio of palmitate and albumin was 3:1) for 1 h followed by adding 100 nmol/L insulin and incubated for another 10 min. 500 μg of total muscle or cell lysis was first immunoprecipitated with IRS-1 antibody, and

immunoblotted with p-Tyr, IRS-1 and p85 antibodies respectively. 20 μg of total muscle or cell lysis was immunoblotted with p-Akt/Akt antibodies. **(B)-(D) The quantitative analysis of western blot results.** The protein bands were qualified. A value of 1 was assigned to the control. Data were means ± SE (n=3), *$p<0.05$ and **$p<0.01$ vs. con by t-test.

Figure S2

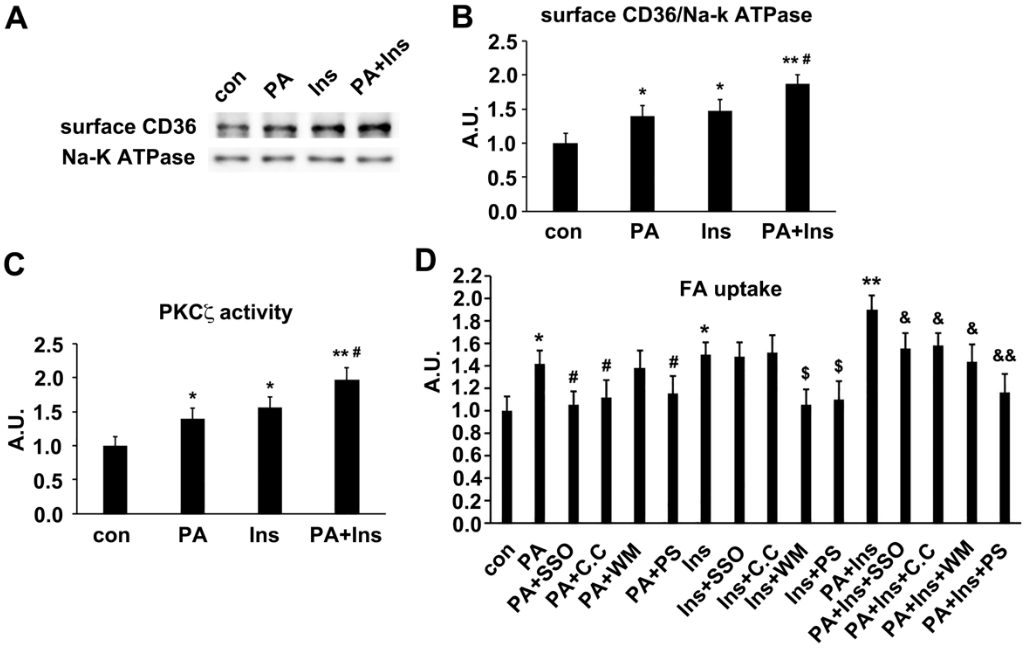

Figure S2. Exclusion of insulin action during the process of PA stimulation. (A) Surface CD36 detection. Cells were left untreated or treated with 500 μmol/L PA (mol ratio of palmitate and albumin was 3:1) for 1 h; or cells were treated with 100 nmol/L insulin for 10 min; or cells were first treated with PA for 50 min followed by adding insulin and incubation for another 10 min. The total protein was extracted and immunoblotted with indicated antibodies. **(B) The quantitative analysis of western blot results.** The protein bands in FigureA were quantified. A value of 1 was assigned to the control condition. Data are means ± SE (n=3), **p*<0.05, ***p*<0.01 vs. con, $^{\#}p<0.05$ vs. PA or Ins, by t-test. **(C) PKCζ activity assay.** Cells were treated in means as shown in part A. PKCζ activity was measured using PKCζ immunoprecipitates from 500 μg of whole cell lysates, as described in Materials and Methods. Data were means ± SE (n=3), **p*<0.05, ***p*<0.01 vs. con, $^{\#}p<0.05$ vs. PA or Ins, by t-test. **(D) FA uptake**

assay. Cells were treated with or without 500 μmol/L PA for 1 h, or 100 nmol/L insulin for 10 min, or PA for 50 min followed by adding insulin and incubation for another 10 min; or cells were first treated with 200 μM SSO, 20 μM C.C, 100 nmol/L WM, 10 μmol/L PS respectively for 20 min, followed by adding PA for 1 h, or insulin for 10min; or PA 50 min plus insulin 10 min. FA uptake was then measured. Data were means ± SE (n=3), $^{*}p<0.05$ and $^{**}p<0.01$ vs. con, $^{\#}p<0.05$ vs. PA, $^{\$}p<0.01$ vs. Ins, $^{\&}p<0.05$ and $^{\&\&}p<0.01$ vs. PA+Ins, by t-test.

Figure S3

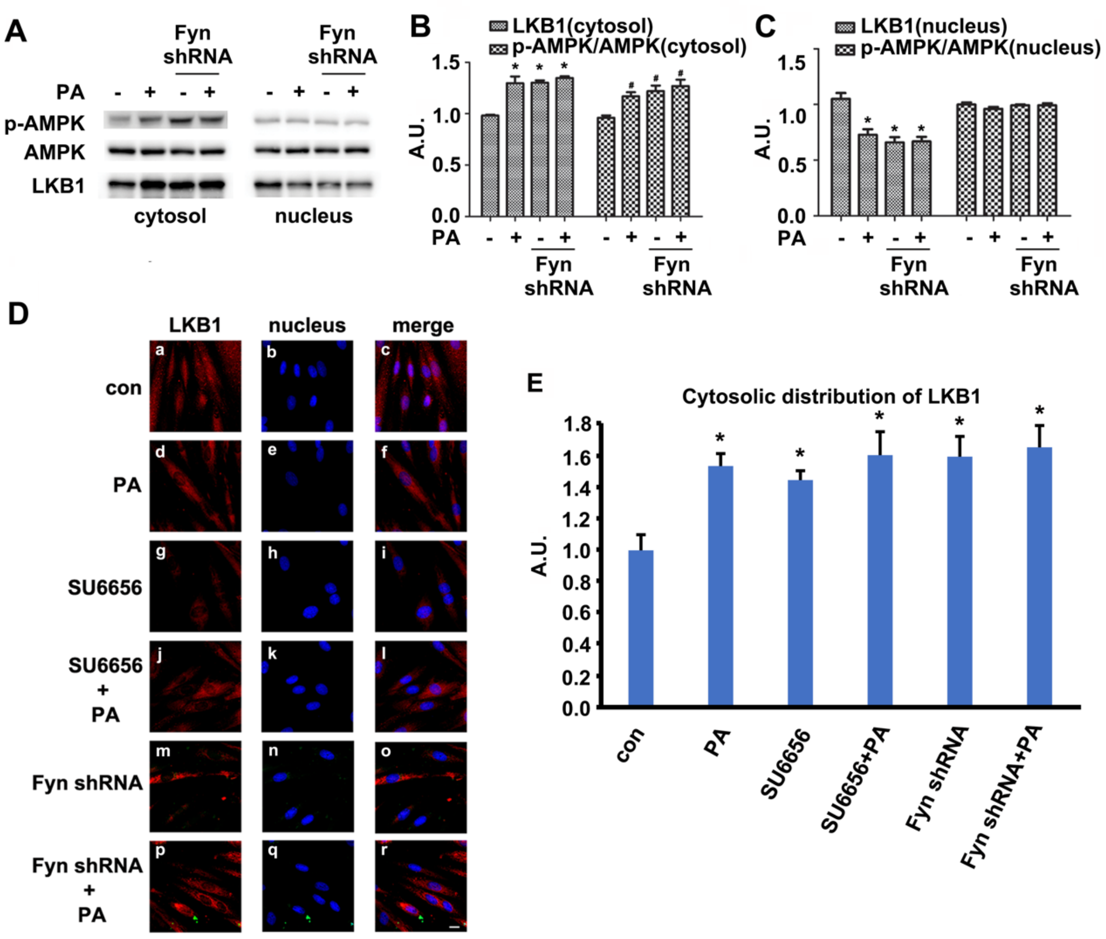

Figure S3. Effect of Fyn on PA-induced LKB1 distribution. (A) Effect of Fyn knockout on LKB1 distribution and AMPK activation. Cells were first transfected with shRNAs and maintained for 48 h. Cells were then left untreated or treated with 500 μmol/L PA for 1 h. Cytosolic and nuclear proteins were immunoblotted with indicated antibodies. **(B)** and **(C) The quantitative analysis of western blot results.** The protein bands in FigureA were quantified. A value of 1 was assigned to the control. Data were means ± SE (n=3), **p*<0.05 vs. con of cytosol or nucleus LKB1, #*p*<0.05 vs. con of cytosol of p-AMPK/AMPK, by t-test. **(D) Fyn was involved in PA-induced LKB1 distribution.** Cells were treated with 500 μmol/L PA for 1 h; or cells were pre-

treated with 10 μmol/L SU6656 for 20 min followed by adding 500 μmol/L PA and further 1 h incubation; or cells were transfected with shRNAs and maintained for 48 h before further 500 μmol/L PA addition and 1 h incubation. LKB1 (red) and nucleus (blue) were stained with LKB1 antibody and DAPI respectively. Bar, 10 μm. The images were representative of three experiments. **(E) Cytosolic LKB1 analysis.** After immunostaining, the signal of LKB1 in whole cell area and nucleus region were scanned with Image J and analyzed with GraphPad Prism software. Data were means ± SE (n=3), *$p<0.05$ vs. con by t-test.

Figure S4

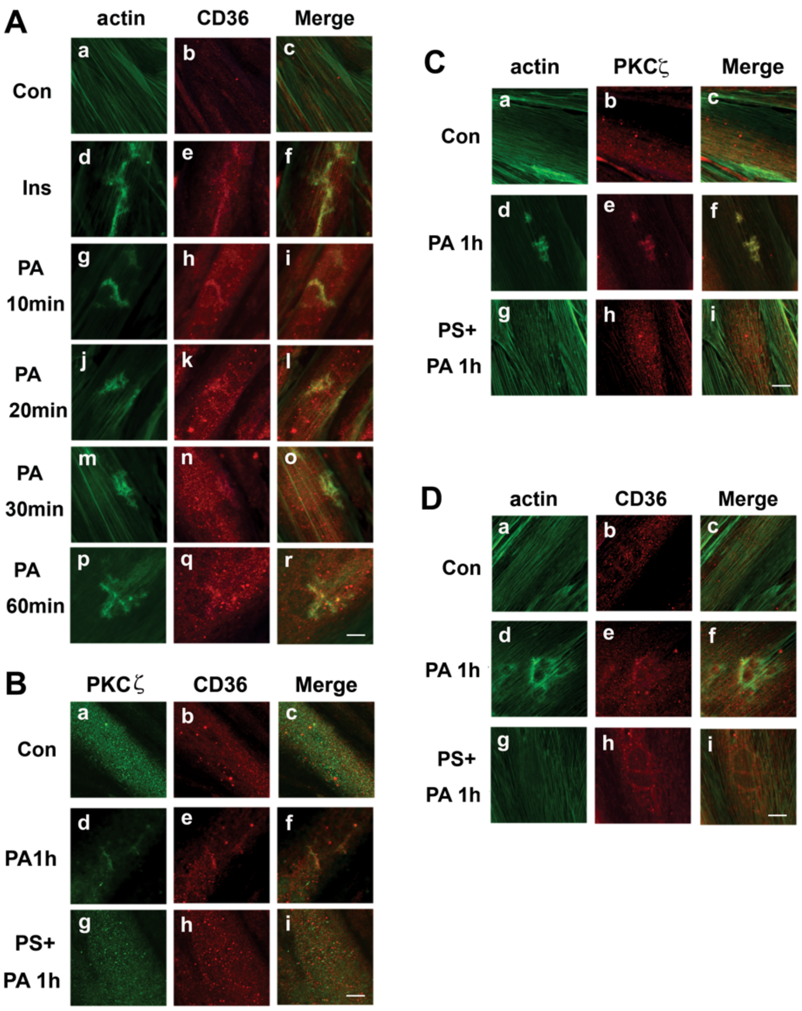

Figure S4. Co-localization of CD36 with PKCζ or actin at dorsal surface of the cells. (A) Colocalization of actin with CD36 after PA treatment. Cells were treated with 100nmol/L insulin for 10 min or with 500 μmol/L PA as indicated. Cells were then stained to examine actin (green) and CD36 (red). Bar, 10 μm. The images were representative of three experiments. **PS blocked PA-induced co-localization of (B) PKCζ with CD36**; and **(C) PKCζ with actin;** and **(D) actin with CD36**. Cells were left untreated or treated with 500 μmol/L PA for 1 h; or cells were pre-treated with 10 μM PKCζ inhibitor (PS) for 20 min followed by further 500 μmol/L PA addition and 1

h incubation. Cells were stained to examine the co-localization between PKCζ (green) and CD36 (red) as well as relationship between actin (green) and PKCζ/CD36 (red). Bar, 10 μm. The images were representative of three experiments.

Figure S5

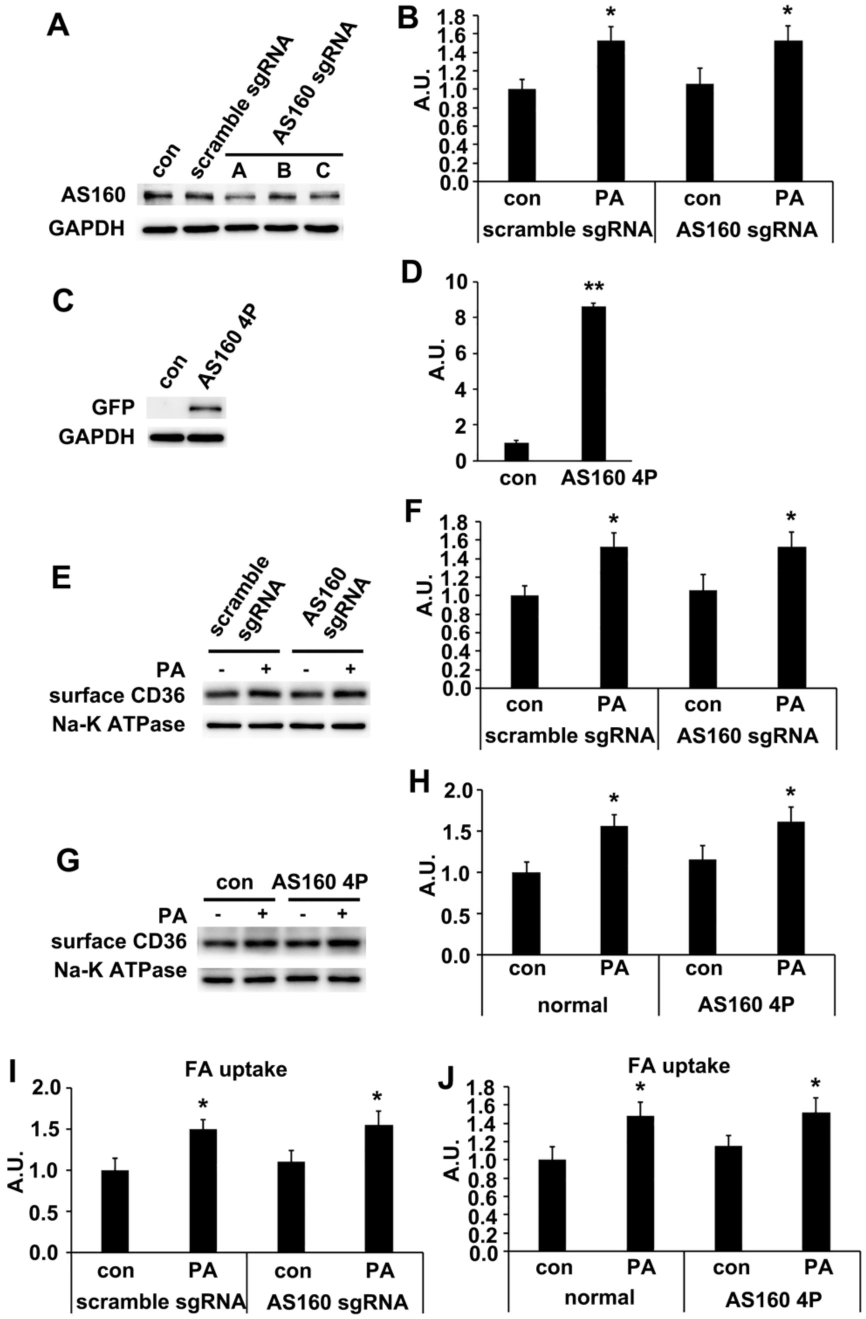

Figure S5. Effect of AS160 on PA-induced CD36 translocation and FA uptake. Assessment of AS160 sgRNAs (A) and AS160 4P overexpression (C). Cells were transfected with plasmids containing sgRNAs or AS160 4P-GFP and maintained for 48 h. Whole cell lysis protein was immunoblotted with indicated antibodies. **Surface**

CD36 detection after AS160 knockdown (E) or upon AS160 4P overexpression (G). Cells were transfected with plasmids containing scramble sgRNA/AS160 sgRNA A (5'-CCGATTGCCCTAGCGCAGCC-3') or AS160 4P and maintained for 48 h. Cells were then treated with or without PA for 1 h. Proteins were examined as indicated. **(B), (D), (F) and (H) The quantitative analysis of western blot results for (A), (C), (E) and (G) respectively.** The protein bands were qualified. A value of 1 was assigned to the control. Data were means ± SE (n=3), *p<0.05 and **p<0.01 vs. con by t-test. **(I) and (J) FA uptake measurement.** Cells were first transfected with sgRNA A or AS160 4P and maintained for 48 h. Cells were then treated with or without PA for 1 h. FA uptake was measured thereafter. Data were means ± SE (n=3), *p<0.05 vs. con by t-test.

Figure S6

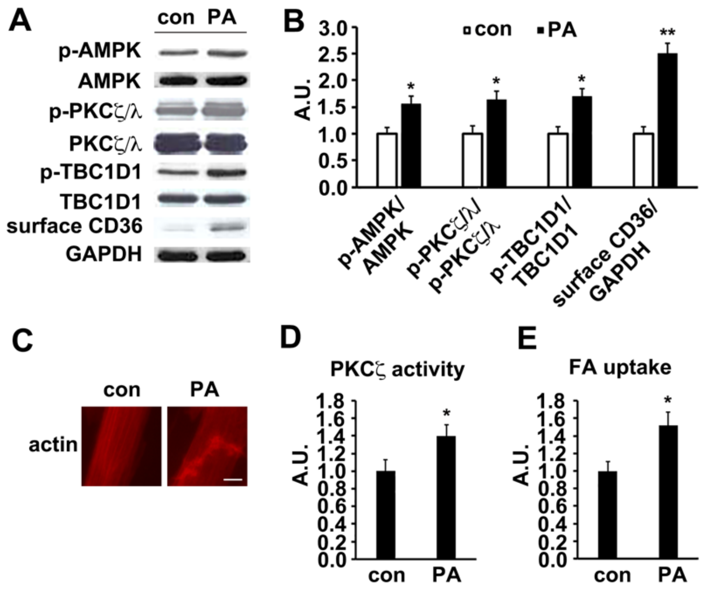

Figure S6. The effect of PA on CD36 relocation via dual modulation of PKCζ and TBC1D1 were tested in C2C12 skeletal muscle cells. (A) Effect of PA on kinase activation and surface CD36 distribution. Differentiated cells were treated with or without 500 μmol/L PA for 1 h. Whole cell lysis and surface proteins were immunoblotted with indicated antibodies. **(B) The quantitative analysis of western blot results.** The protein bands in (A) were qualified. A value of 1 was assigned to the control. Data were means ± SE (n=3), *p<0.05 and **p<0.01 vs. con by t-test. **(C) Immunostaining of actin after PA treatment.** Cells treated with 500 μmol/L PA for 1 h were fixed and stained for actin (red), Bar, 10 μm. The images were representative of three experiments. **(D) Measurement of PKCζ activity.** PKCζ activity was measured using its immunoprecipitates from 500 μg of whole cell lysates. Data were means ± SE (n=3), *p<0.05 vs. con by t-test. **(E) FA uptake assay.** Cells were treated with 500 μmol/L PA for 1 h, FA uptake was then measured. Data were means ± SE (n=5), *p<0.05 vs. con by t-test.

Figure S7

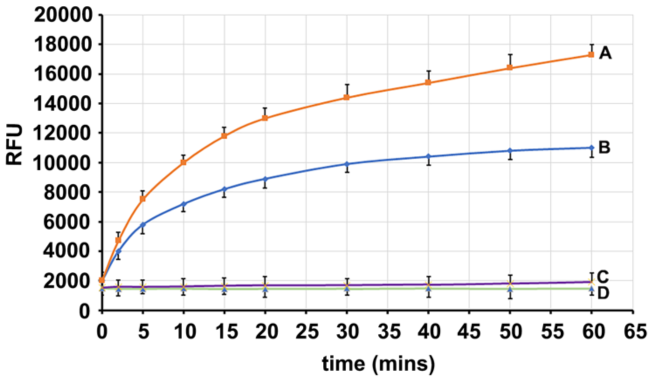

Figure S7. Effect of PA-induced FA uptake. Cells were left untreated or treated with 500 µmol/L PA for 1 h, 0.1 mmol/L BODIPY-FA was then added for FA uptake measurement. For the blank wells, cells were treated with 0.1mmol/L BODIPY-FA+500 µmol/L PA or 0.1mmol/L BODIPY-FA+10 µmol/L Cytochalasin B (CB) to measure FA uptake. Traces A to D corresponded to PA, control, blank 1 (BODIPY-FA+PA) and blank 2 (BODIPY-FA+CB) respectively.
